# Vanadium in Biological Action: Chemical, Pharmacological Aspects, and Metabolic Implications in Diabetes Mellitus

**DOI:** 10.1007/s12011-018-1540-6

**Published:** 2018-10-22

**Authors:** Samuel Treviño, Alfonso Díaz, Eduardo Sánchez-Lara, Brenda L. Sanchez-Gaytan, Jose Manuel Perez-Aguilar, Enrique González-Vergara

**Affiliations:** 1 Facultad de Ciencias Químicas, Benemérita Universidad Autónoma de Puebla, 14 Sur y Av. San Claudio, Col. San Manuel, C.P. 72570 Puebla, PUE Mexico; 2 Centro de Química, ICUAP, Benemérita Universidad Autónoma de Puebla, 14 Sur y Av. San Claudio, Col. San Manuel, C.P. 72570 Puebla, PUE Mexico

**Keywords:** Vanadium, Biological action, Metabolic aspects, Metabolic implications, Metallopharmaceuticals, Diabetes mellitus

## Abstract

Vanadium compounds have been primarily investigated as potential therapeutic agents for the treatment of various major health issues, including cancer, atherosclerosis, and diabetes. The translation of vanadium-based compounds into clinical trials and ultimately into disease treatments remains hampered by the absence of a basic pharmacological and metabolic comprehension of such compounds. In this review, we examine the development of vanadium-containing compounds in biological systems regarding the role of the physiological environment, dosage, intracellular interactions, metabolic transformations, modulation of signaling pathways, toxicology, and transport and tissue distribution as well as therapeutic implications. From our point of view, the toxicological and pharmacological aspects in animal models and humans are not understood completely, and thus, we introduced them in a physiological environment and dosage context. Different transport proteins in blood plasma and mechanistic transport determinants are discussed. Furthermore, an overview of different vanadium species and the role of physiological factors (i.e., pH, redox conditions, concentration, and so on) are considered. Mechanistic specifications about different signaling pathways are discussed, particularly the phosphatases and kinases that are modulated dynamically by vanadium compounds because until now, the focus only has been on protein tyrosine phosphatase 1B as a vanadium target. Particular emphasis is laid on the therapeutic ability of vanadium-based compounds and their role for the treatment of diabetes mellitus, specifically on that of vanadate- and polioxovanadate-containing compounds. We aim at shedding light on the prevailing gaps between primary scientific data and information from animal models and human studies.

## Introduction and Background

The element vanadium is considered the twice discovered element due to the circumstances by which Andrés Manuel Del Río, a Spanish-Mexican mineralogist, first reported it in 1801 [1]. Renowned personalities such as Lavoisier, Delhuyar, Von Humboldt, Berzelius, and Whöler were involved directly or indirectly in its discovery. In 1791, Del Río was an assistant at Lavoisier’s laboratory; unfortunately, on November 8, 1793, Lavoisier was arrested and Del Río fled from Paris to England as he was afraid of being arrested. In that same year, Fausto Delhuyar, co-discoverer of tungsten, offered him the chair of chemistry at the newly organized Royal School of Mines in Mexico City. However, he preferred the chair of mineralogy, so he was appointed the chairman and arrived in Mexico in December 1794. Among his duties, Del Río had the task of organizing the mineral collection at the College of Mines and carrying out chemical analysis of newly discovered minerals. In 1801, in a mineral called brown lead from La Purísima del Cardenal mine near Zimapán in what is now the Mexican State of Hidalgo, Del Río discovered a new element. Initially, it was called *panchromium* and later *erythronium* due to the red color of its salts with alkaline and alkaline earth metals. The first publication of this new element appeared in a Spanish journal in 1802 [2]. To popularize his discovery, Del Río completed a full paper describing his experiment and conclusions and addressed it to the French chemist Jean Antoine Chaptal. Unfortunately, the ship carrying it wrecked in Pernambuco, Brazil. In April 1803, a graduate student from the Freiberg Mining Academie, where Del Río also graduated from, arrived in Mexico. This student was Alexander Von Humboldt. They resumed their friendship, and Del Río told Humboldt about his newly discovered element. Though skeptical, Humboldt agreed to take Del Río’s new paper and samples of brown lead to describe the discovery of erythronium in more detail on his way back to Europe. However, news about the discovery of chromium by Vauquelin in 1797 did not reach Mexico till 1803 and its resemblance to chromium compounds convinced Del Río that his discovery was chromium. After his arrival in Paris in August 1804, Humboldt gave a sample of brown lead to Hippolyte-Victor Collet-Descotils at the Institut de France. He analyzed the sample and at the conclusion of his report, Collet-Descotils wrote [3], “The experiments that I have reported appear to me sufficient to prove that this ore contains nothing of a new metal.” Unfortunately, Humboldt accepted Collet-Descotils’ conclusion that erythronium was chromium, and Del Río’s paper was never published. In 1831, Nils Gabriel Sefström discovered a new element from an ore mined in Taberg, Småland, Sweden. He named the element vanadium after Vanadis, one of Freya’s names, the Norse goddess of love and beauty. Friedrich Wöhler was simultaneously reinvestigating the composition of brown lead from Zimapán, working with a sample that Humboldt had given him. Sefström gave some vanadium pentoxide to J. J. Berzelius, who demonstrated that the new element was not uranium. Berzelius sent some of the vanadium pentoxide to Wöhler, who conclusively showed that vanadium was identical to Del Río’s erythronium. At a session of the French Academy of Science on February 28, 1831, Alexader Von Humboldt described the discovery of vanadium, granting equal credit to Sefström, Wöhler, and Del Río [4].

Nowadays, the chemistry of vanadium is currently being tested to be used in electrochemical storage systems such a vanadium redox flow batteries. Also, as a photographic developer, drying agent in various paints and varnishes, reducing agent, and the production of pesticides, as well as the black dyes, inks, and pigments that are employed by the ceramics, printing, and textile industries [5–7].

**Fig. 1:**
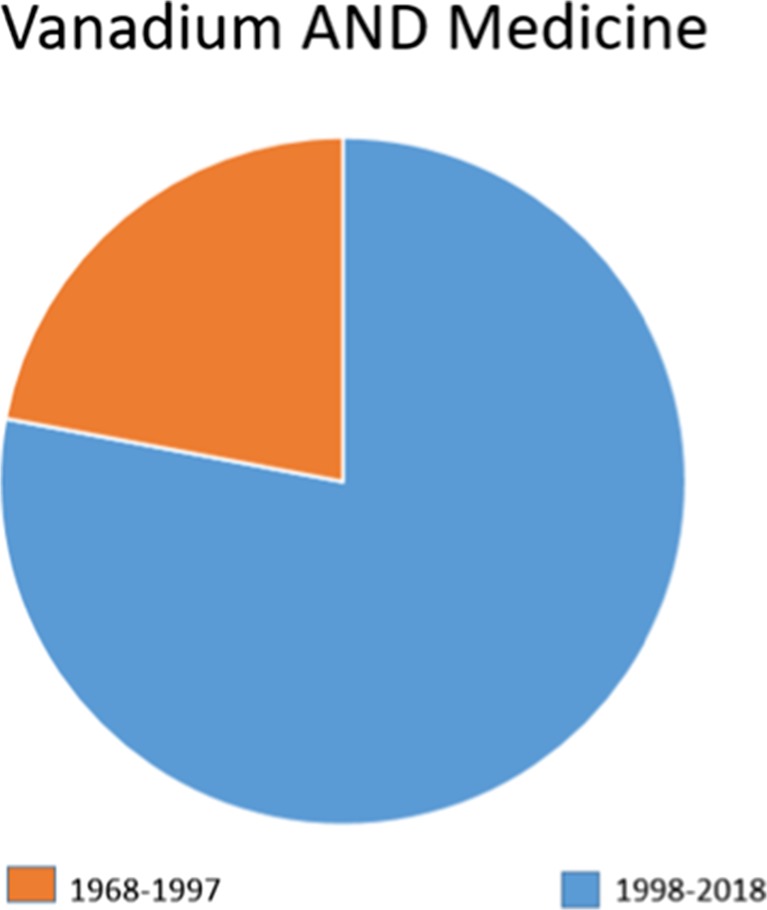
PubMed entries for vanadium and medicine in the last 50 years

It is, however, in the biological sciences, that the unique vanadium properties can be exploited. To date, the pharmacological behavior of several vanadium compounds has shown very promising results, which has prompted their study from numerous groups around the world. A brief search in PubMed displays more than 8000 reports in which vanadium compounds show an application in medicine or public health problems.

(see Fig. 1). Additionally, more than 4000 patents of vanadium compounds have been filed for their use as anti-parasitic, anti-viral, antibacterial, anti-thrombotic, anti-hypertensive, anti-atherosclerotic, spermicidal, anti-HIV, and anti-tuberculosis; however, the majority of the patents focus in their use as anti-cancer and anti-diabetic drugs.

This review aims to give an update of the relevant aspects of vanadium biochemistry with an emphasis in metabolic actions and its corresponding metabolic implications for the development of new and potentially useful vanadium-based pharmacological compounds.

## Environmental Exposition and Toxicology of Vanadium

Vanadium is the 22nd most abundant element on earth (0.013% *w*/*w*), and it is widely distributed in all organisms. In humans, the vanadium content in blood plasma is around 200 nM, while in tissues is around 0.3 mg/kg and mainly found in bones, liver, and kidney. In vertebrates, vanadium enters the organism principally via the digestive and respiratory tracts through food ingestion and air inhalation [8, 9]. The estimated daily dietary intake in the USA is 10 to 60 μg/day, where unprocessed foods have variable amounts up to 10 ppb, but not exceeding 1 μg/g from animal or plant source foods. Likewise in other countries, the dietetic vanadium concentrations of dry weight fluctuate largely, e.g., Brazil (21.6–54.2 μg/g), Iran (32.6–135 μg/g), Italy (12.1–154 μg/g), Spain (7.8–315 μg/g), Thailand (7.7–30.5), and Turkey (18.7–78.9) [10–12]. Also, vanadium is found in potable water in concentrations around 1 μg/L; thus, its intake by this source depends on the daily ingested volume [13]. Therefore, the typical daily dose consumed by humans corresponds to 10–30 μg of vanadium per day; however, most of the dietary vanadium is usually excreted in the feces, meaning that the vanadium accumulation in the body does not constitute a potential hazard [14–17].

### Vanadium Entrance via the Respiratory System

Ambient air concentrations of vanadium are naturally low. Rural areas present vanadium concentrations around 0.8–1.2 ng/m^3^, but urban areas tend to present higher concentrations (3.0–3.7 ng/m^3^). In urban areas, vanadium contents in the breathing air can go up to 103 ng/m^3^, namely two to three orders of magnitude more than in rural areas. In places with high density of oil-fired power plants using vanadium-rich residual fuel oil, average vanadium air concentrations can go up to 620 ng/m^3^ [9, 13]. Based on occupational exposure studies, human experimental studies, and studies in laboratory animals, the respiratory tract is one of the primary targets of vanadium toxicity following inhalation exposure. Adverse respiratory effects have been reported in humans and animals exposed to vanadium compounds at concentrations much higher than those typically found in the environment. Although the available data in humans is limited, signs of airway irritation (e.g., coughing, wheezing, and sore throat) have been reported in subjects acutely exposed to 0.6 mg vanadium/m^3^ and in workers exposed to vanadium pentoxide (V_2_O_5_) dust. The effects persist for days or weeks after exposure termination and are often not associated with alterations in lung function [13, 18–23]. On the other hand, a variety of lung lesions including alveolar/bronchiolar hyperplasia, inflammation, and fibrosis have been observed in rats and mice exposed to V_2_O_5_, VOSO_4_, or NaVO_3_, where the lesions severity are in concordance to concentration and duration of the exposition. The lung effects have been observed following acute exposure to 0.56 mg vanadium/m^3^ and chronic exposures to 0.28 mg vanadium/m^3^ and have been observed after 2 days of exposure. Longer duration exposures also result in inflammation and hyperplasia in the larynx and hyperplasia in the nasal goblet cells. The histological alterations result in restrictive impairments of lung function and respiratory distress is observed at vanadium pentoxide concentrations ≥ 4.5 mg vanadium/m^3^ [24–28]. The minimal risk level (MRL) that is defined by the Agency for Toxic Substances and Disease Registry (ATSDR) is an estimate of the daily human exposure to a substance that is likely to be without an appreciable risk of adverse effects. In the case of vanadium inhalation in acute, intermediate, and chronic exposition, the dosage of the no-observed-adverse-effect level (NOAEL) and lowest-observed-adverse-effect level (LOAEL) is presented in Table 1.

**Table 1:** Minimal risk level (MRL) for vanadium inhalation and oral ingestion

Acute-duration inhalation MRL	0.0008 mg of V_2_O_5_/m^3^/14 days [24]
No-observed-adverse-effect level (NOAEL)	0.34–0.56 mg of vanadium/m^3^/13 days [24, 29]
Lowest-observed-adverse-effect level (LOAEL)	0.56 mg of vanadium/m^3^ [24, 29]
Intermediate-duration inhalation MRL	4.4 mg of V_2_O_5_/m^3^ for 6 h/day, 5 days/week for at least 4 weeks [29, 30]
No-observed-adverse-effect level (NOAEL)	0.56 mg of vanadium/m^3^ [29, 30]
Lowest-observed-adverse-effect level (LOAEL)	4.5 mg of vanadium/m^3^ in males [30] 2.2 mg vanadium/m^3^ in females [30]
Chronic-duration inhalation MRL	0.0001 mg of V_2_O_5_/m^3^ for 6 h/day, 5 days/week for at least 1 year [29]
No-observed-adverse-effect level (NOAEL)	Undefined
Lowest-observed-adverse-effect level (LOAEL)	≥ 0.56 mg of vanadium/m^3^ [29]
Acute-duration oral MRL	0.009 mg of vanadium/kg/day [13]
No-observed-adverse-effect level (NOAEL)	0.2 mg of vanadium/kg/day [31, 32]
Lowest-observed-adverse-effect level (LOAEL)	0.35 mg of vanadium/kg/day [31, 33–35]
Intermediate-duration oral MRL	0.01 mg of vanadium/kg/day for 15–364 days [36–38]
No-observed-adverse-effect level (NOAEL)	0.12 mg of vanadium/kg/day for 365 days [37]
Lowest-observed-adverse-effect level (LOAEL)	1.18 mg of vanadium/kg/day [36]
Chronic-duration oral MRL	Undefined
No-observed-adverse-effect level (NOAEL)	Undefined
Lowest-observed-adverse-effect level (LOAEL)	Undefined

### Vanadium Entrance via Digestive System

The digestive tract is another access way for vanadium. Studies in animals have shown that less than 5% of the ingested vanadium is absorbed while the rest is excreted via the feces. In concordance, human studies reported that vanadium is poorly absorbed (0.2% to 1.0%). It is clear that fasting, dietary composition, and speciation may affect absorption [39–44]. Although subjects that consumed doses of 7.8–10 mg vanadium/day/2 weeks do not show adverse symptoms, higher doses (14–42 mg vanadium/day/2 weeks) cause gastrointestinal problems including abdominal discomfort, irritation, cramping, diarrhea, nausea, and vomiting [45, 46]. There is no evidence for extrapolation of the daily dose expressed per unit of body weight. Using the NOAEL of 0.12 mg vanadium/kg/day and an uncertainty factor of 10 for human variability, the MRL would be 0.01 mg vanadium/kg/day. The LOAEL dose has been identified a minimal value of 1.18 mg vanadium/kg/day. Dividing the LOAEL dose by an uncertainty factor of 300 (3 for the use of a minimal LOAEL, 10 for the animal to human extrapolation, and 10 for human variability), results in an MRL of 0.004 mg vanadium/kg/day are obtained. However, the Fawcett study was selected as the basis for the intermediate-duration oral MRL because this was given to an MRL based on a reliable human study [33]. Thus, the intermediate-duration oral MRL is 0.01 mg vanadium/kg/day. To the best of our knowledge, no studies of the chronic toxicity of vanadium in humans have been done. Moreover, as a consequence, the chronic-duration oral MRL for humans has not been established, because recent reports do not exist (Table 1).

Meanwhile, significant increases in reticulocyte levels in peripheral blood and polychromatophilic erythroblasts in the bone marrow were observed in rats exposed to a dose of 27.72 mg vanadium/kg/day for 2 weeks [31]. The dose of 7.5–8.4 mg vanadium/kg/day during gestation reported developmental effects in the offspring of rats and mice that included decreases in fetal growth and increases in resorption anomalies as well as gross, visceral, and skeletal, malformations [34, 35, 47]. Thus, in this case, the identified LOAEL dose in the animal for developmental effects corresponds to 7.5 mg vanadium/kg/day [35]. The NOAEL dose has been established at 0.2 mg of vanadium/kg/day for an acute-duration oral MRL. Long-term vanadium treatments observed significant decreases in erythrocyte counts in rats exposed to 1.18 mg vanadium/kg/day in the form of ammonium metavanadate in drinking water during 4 weeks [32] and a decrease in hemoglobin which is compensated with reticulocyte increase in peripheral blood [32, 48–53]. However, previous intermediate-duration studies did not found significant alterations in doses up to of 9.7 mg vanadium/kg/day [36, 37]. Notably, the consumption of 1.72 mg vanadium/kg/day showed impaired performance on neurobehavioral tests (open field and active avoidance tests) in rats exposed to sodium metavanadate for 8 weeks [38]. BALB/c mice (4 weeks old) administered with 3 mg vanadium/kg/day (sodium metavanadate), thrice a week for 3, 6, 9, 12, 15, and 18 months, showed astrocytic and microglial activation after 6 months. Also, the cortical pyramidal cells showed morphological alterations including pyknosis, cell clustering, loss of layering pattern and cytoplasmic vacuolation, dendritic arborization loss of the pyramidal cells of the dorsal hippocampal CA1 region, and the Purkinje cell layer lost [54].

## Absorption and Speciation In Vivo

**Fig. 2:**
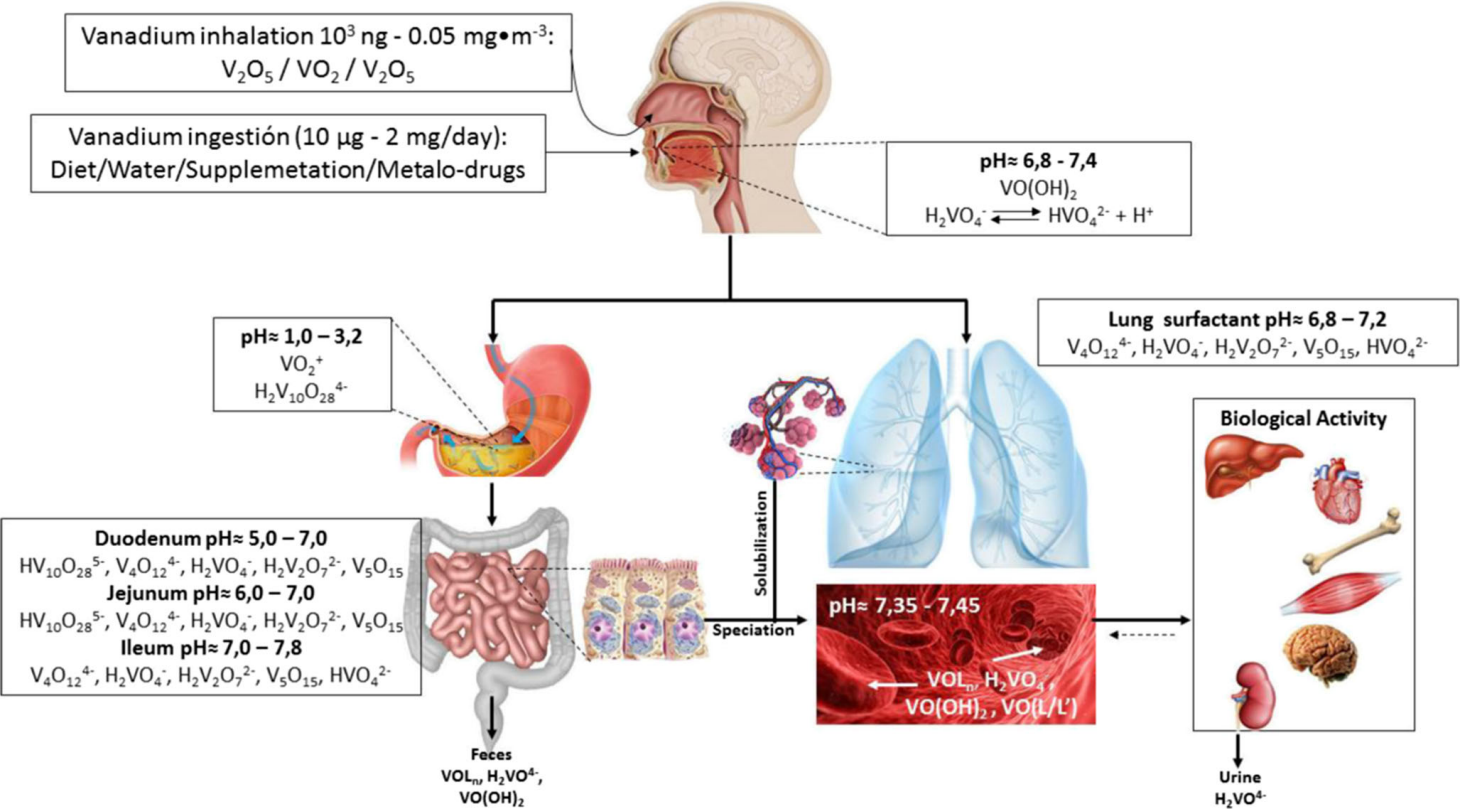
Speciation and solubilization in pH dependence in different body cavities and tissues

There are two main routes for the absorption of vanadium in the organism which, depending on the dose, can constitute health hazards: breathing and ingestion. Lungs constitute the main site of entry for environmental exposure of vanadium through breathing (Fig. 2). The size of vanadium-containing particles and the solubility of vanadium compounds are important factors in the determination of vanadium absorption rate in the respiratory tract. For instance, lung clearance of the insoluble vanadium pentoxide is relatively rapid in animals after acute exposure, but substantially slower after chronic exposure. This occurs because over time the metal is slowly deposited in the lungs and tends to remain there. Soluble compounds are also partly absorbed, but the extent of absorption in the respiratory tract has not been determined. After breathing vanadium-containing compounds, vanadium acts directly on human the bronchial smooth muscle promoting the release of Ca^2+^ from the intracellular store via the production of inositol phosphate second messengers and inhibition of Ca^2+^-ATPase, causing spasms [55]. Wang et al. described the mechanism of multiple reactive oxygen species induced by vanadium absorption that involves activation of an NADPH oxidase complex and the mitochondrial electron transport chain, with hydrogen peroxide playing a major role in lung inflammation and apoptosis [56]. The free radical redox cycle of vanadium was studied in rat lungs and involves a one-electron redox cycle in lung biomembranes and reduction of vanadium V to vanadium IV (i.e., vanadium speciation), which initiates lipid peroxidation and possibly contributes to pulmonary toxicity [57]. Ingestion is the other important via vanadium absorption. Based on the estimated daily vanadium intake and levels in urine and feces, less than 5% of ingested vanadium is intestinally absorbed (Fig. 2). One of the first studies of vanadium absorption used the radioisotope ^48^V as a tracer and found that about 15% of vanadium (as Na_3_VO_4_) in a single bolus is absorbed [58]. However, that value is well above of the 1–3% of absorption that most studies have found [59, 60]. Also, it has been estimated that no more than 1% of vanadium contained in the diet is absorbed [40]. Human studies agree well with animal studies and have stated that only between 0.13 and 0.75% of ingested vanadium (i.e., ammonium metavanadate) is retained in the body [41]. Oral ingestion of vanadium mainly involves two species: vanadates (HVO_4_^2−^, oxidation state + 5; V^5+^) present in drinking water and vanadyl (VO^2+^, oxidation state + 4; V^4+^). V^5+^ compounds are partially reduced in the stomach and later precipitated in the slightly alkaline medium of the intestines to form sparingly soluble VO(OH)_2_ (Fig. 2) [9, 18]. On the other hand, HVO_4_^2−^ is more easily taken up in the gastrointestinal tract and is absorbed 3 to 5 times more effectively than VO^2+^. Thus, the speed at which the vanadium compounds are transformed in the organism and the species in which it transforms effectively affects the percentage of ingested vanadium which is absorbed [61].

Depending on the surrounding solvent, different of vanadium species are favored. [62–65]. Each one of these species has different chemical properties and therefore different biological responses in fluids, tissues, or cells [15, 62, 66–75] and can form different complexes. Moreover, the speciation of vanadium compounds and salts is also sensitive to their conditions and environment, and as a result, their chemical bioprocessing is essential to understand their mode of action [62, 68]. Several works have demonstrated the degree of transformation or speciation that vanadium compounds suffer under different environmental and biological conditions [76]. Routine methods used for measurement of speciation in aqueous solution include nuclear magnetic resonance (NMR) and electron paramagnetic resonance (EPR) spectroscopy, UV–Vis spectroscopy, potentiometry, and electrochemistry [65, 76–78].

Aqueous vanadyl can exist in both cationic and anionic forms [66, 74, 79]. Cationic species tend to form at acidic pH, while anionic species forms at neutral and basic pH (Fig. 2). Little is known about the speciation of aqueous vanadyl at neutral pH, mainly because at this pH there is no electron paramagnetic resonance (EPR) signal, presumably because of the dimerization/oligomerization of the vanadyl species or oxidation to V^5+^ [68, 80]. Oxidation states of vanadium are of paramount importance in the development of new compounds with biological applications because of the impact it will have in its pharmacological and pharmacokinetic properties. For instance, it has been considered that V^4+^ forms tend to form stable coordination complexes with ligands, and the equilibration of these systems is slower than those for V^5+^ systems [64, 80, 81]. However, recent work has demonstrated that this perception does not hold for all types of ligands and that there are V^5+^ complexes which pharmacological characteristics render more potent anti-diabetic agents than V^4+^ complexes [71, 72, 82, 83]. The vanadate HVO_4_^2−^ ion has rich chemistry (in part due to its pK_a_ of 12) that gives rise to a number of species that can be formed at different pH. At pH below of 12, two HVO_4_^2−^ ions can condense and release water to form the vanadate dimer, V_2_O_7_^4−^, which in turn can be protonated in a more acidic medium. A further increase in acidity to near neutral conditions promotes the formation of higher oligomers. Under those conditions, the predominant species are tetramer or pentamer cyclic oligomers, V_4_O_12_^4−^ or V_5_O_15_^5−^. Other oligomers that are normally found as minor components of an equilibrated solution are the cyclic hexamer and the linear trimer, tetramer, and hexamer species [84–86]. The relative distribution of the different species concentrations depends on the total vanadate concentration in such a way that compounds of lower nuclearity are favored at low concentrations. At pH of 6 or below and concentrations of 0.2 mM and above, the vanadium decamer (decavanadate) is formed and it is the predominant species; however, a mixture of decavanadate, monoprotonated and diprotonated decavanadate, and small amounts of tetravanadate as well as free vanadate are present. Furthermore, the protein interplay into cell leads to decavanadate stabilization, thus suggesting that V10 interacts with specific locations within these (e.g., alkaline phosphatase, adenylate kinase, P-type ATPases, ABC ATPases, F-actin, myosin ATPase, and ribonuclease) protecting the decameric species against conversion to the structurally and functionally distinct lower oxovanadates (vanadium monomer, dimer, or tetramer) [86, 87]. Unlike other vanadate oligomers, this oligomer undergoes successive protonation reactions with an increase in acidity, going from a charge of − 6 to − 3, being the − 4 and − 5 anions the predominant forms. Very strong acidic conditions (i.e., below a pH = 2) will cause the decavanadate to be replaced by the cationic species, [VO_2_(H_2_O_4_)]^+^ (often referred to as VO_2_^+^). Because of its high proton stoichiometry compared to the other vanadate derivatives, the cation is frequently the only compound in a significant concentration in solution under strongly acidic conditions, even in the presence of strong-binding ligands. Since vanadium possesses a high ability to change oxidation states or to exchange ligands depending on the environment, the surrounding molecules will have a great impact on the vanadium passage through cell membranes. There is, therefore a necessity to develop vanadium-based drugs containing ligands that protect the compound from speciation to conserve its pharmacological properties and to enhance its absorption. Vanadium speciation is a relevant characteristic of vanadium and impacted by the presence of biological or synthetic chelators, biogenic ligands, or functional carriers [88, 89]. In our laboratory, we work with counter-cations such as ammonium, dimethylaminopyridine, and biguanides (metformin) to stabilize charges and maintain the biological activity [86, 90–92].

## Transport of Vanadium Species in Blood

Vanadium compounds are exposed to diverse environments during their administration before reaching the bloodstream. For instance, these can be solubilized by lung surfactant in alveoli or be exposed to a highly acidic environment in the stomach before suffering biotransformation into the biologically active forms that circulate the blood plasma. Once in the bloodstream, vanadium species bind to serum proteins, particularly transferrin and albumin. Vanadyl displays a strong preference to bind not only proteins but also negatively charged serum molecules of low molecular weight such as citrate, oxalate, lactate, phosphate, glycine, and histidine [93]. At biologically relevant concentrations of vanadyl (i.e., V^4+^ < 5 mM), most of the vanadium in the bloodstream is bound to transferrin, where the V^4+^ ion binds to the same binding site as the Fe^3+^ ion [94]. The presence of a metal binding site in transferrin makes it a more efficient vanadium carrier than albumin; it is well established that vanadium can displace 30–70% of the original iron ion from the transferrin complex [79, 95–100]. Interestingly, it has been shown that even though V^4+^ displays a high affinity for the iron binding site of transferrin, it is the V^5+^ the species capable of binding this protein in the absence of the synergistic anion (e.g., carbonate) that is required for the iron binding [101–103]. Lastly, at higher concentrations, it has been shown that bloodstream V^4+^ can even bind to immunoglobulin G [104–106]. Finally, some vanadyl species with insulin-enhancing properties exhibit a relatively long lifetime in the bloodstream that may allow correlating the vanadium blood content with its binding to the transport protein albumin [107].

In extracellular fluids, vanadium, in the form of vanadate and vanadyl, is either reduced or oxidized respectively, depending on the presence of different redox-active agents. In the case of vanadate, due to the pKa at physiological pH, ionic strength, low concentration, and potential ligands, the V^5+^ ion exist in blood plasma mainly as either H_2_VO_4_^−^ and HVO_4_^−2^, and it is not expected to form oligovanadates [108].

Moreover, regarding the binding of vanadium to one of the main carrier plasma proteins, transferrin, there is a significant amount of experimental evidence indicating the binding of the V^5+^ ion to the same binding site occupied by the Fe^3+^ ion [101, 102, 109–114]. Structural details indicate that vanadate binds to both, the N- and C-terminal sites as VO_2_^+^ where is coordinated by Tyr, His, and Asp residues. Furthermore, it has been found that significant amounts of the V^5+^ ion are bound to transferrin in solutions containing the iron-bound protein, suggesting that V^5+^ could either be located at a different binding site or that it could act as a synergistic anion to the iron binding site [103, 110]. Lastly and along these lines, competition binding experiments in apotransferrin between HCO_3_^−^ and H_2_VO_4_^−^ for the site normally occupied for the synergistic anion concluded that no competition between these two molecules exist and that the V^5+^ ion can form (hydrogen)carbonate-V^5+^ adducts, similarly to those form with phosphate (phosphate-V^5+^) [111].

Albumin plays a major role in the transport of metals in the plasma. Structurally the protein contains two metal binding sites, an N-terminal site (NTS or site I) that specifically binds Cu^+2^ and Ni^+2^ ions, and a multimetal binding site (MBS or site II) that primary binds Zn^+2^ ions but also displays high affinity for Cu^+2^ and Ni^+2^ ions [94, 115–119]. As for the interaction of the protein with vanadium, particularly the VO^2+^ species, studies have identified a high-affinity binding site (VBS1) and at least five relatively low-affinity vanadium binding sites (VBS2) [94, 120, 121]. In this context, competition studies between Zn^+2^ and VO^2+^ ions showed that the latter is bound to albumin at two binding sites, one of them corresponding to the MBS (or VBS1, which primarily binds a Zn^+2^ and thus constitute a metal binding competition site between Zn^+2^ and VO^2+^) and another (VBS2) where no metal binding competition occurs [122]. Additionally, EPR experiments suggest that VO^2+^ ions formed a binary adduct that interacts with residues located at the MBS (VBS1) [103, 122].

Since specific coordination sites like those of transferrin for iron are lacking, albumin form mixed complexes with vanadium giving rise to pharmacologically active species [113, 123]. Therefore, some vanadyl species with the sufficiently long lifetime in the bloodstream exhibit a good capacity to lower plasma glucose in diabetic models associated with its binding to human albumin [107]. Also, CD spectra suggest more than two types of binding sites to albumin, in which at physiological pH, the main (VOL_2_)_n_-albumin species can co-exist with a minority (VOL)_n_-albumin mixed complex [120, 124].

The results obtained for the interaction of V^+5^-albumin complexes are not so clear and straightforward as those obtained for the V^+5^-apo-hTF system. However, different studies agree that the interaction is weak and unspecific, some of them suggesting that the binding sites probably involve surface carboxylic groups. Crans et al. proposed a relation V^+5^-albumin of 1:1 [66]. Heinemann et al. concluded that V^+5^ is bound to albumin in very low concentration (maximum 0.3–0.4%) [125]. Kiss et al. by literature data, estimated a log *K* value of 1.8 ± 0.3 for V^+5^-albumin complexes [68]. Castro et al. showed evidence that some V^+5^ complexes can bind to drug site I by ^1^H saturation transfer difference (STD) NMR spectroscopy and computational docking studies [125, 126].

Additionally, the interaction between vanadium and the serum protein immunoglobulin IgG has been investigated. At physiological conditions, interactions of VO^2+^ in three distinct superficial IgG binding sites, namely 1, 2, and 3, were identified. Interacting features of site 2 resemble those observed in VBS2 of albumin while in site 1 may be the most probable candidate to established interactions with VO^2+^ [12, 104, 127].

The oxygen carrier protein, hemoglobin (Hb), has also been involved in the vanadium bloodstream transport. Since the erythrocyte environment presents a reducing environment for vanadium, mainly driven by glutathione, the investigation involved the V^4+^ oxidation state exclusively [88, 127–133]. Most of the experimental studies indicate that, inside the erythrocytes, the VO^2+^ ion is mainly bound to hemoglobin [88, 127–129, 132, 133], although possible competition for vanadium may arise from some intracellular bioligands [129]. Utilizing EPR spectroscopy, three non-specific pH-dependent Hb binding sites for VO^2+^ have been identified, namely α, β, and γ sites [88]. The vanadium α binding site is only composed of carboxylate groups (from Asp and Glu) while β and γ sites also contain imidazole groups (from His) as part of the vanadium coordination sphere. Notably, at pH 7.4, only the β and γ sites in Hb seem to be occupied. The stability constant (binding constant) for the interaction between VO^2+^ and plasma blood proteins is transferrin >> hemoglobin ≈ immunoglobulin G > albumin [123]. However, it must be taken into account the type of vanadium complexes and their decomposition grade, saturation, speciation, and excretion to each ligand.

### Vanadium Compounds Species at Physiological Conditions

Although synthetic inorganic chemistry has developed different kinds of oligovanadates, by considering the different physiological conditions (e.g., absorption environment, concentration, pH, ionic strength) it is unlikely the vanadate oligomers can last inside the body for long periods of time based on their thermodynamic instability. At pH ≈ 7, the only vanadate of relevance is the monovanadate H_2_VO_4_^−^ compound; however, at higher vanadate concentrations, the formation of tetravanadates becomes more feasible.

Decavanadate (V_10_O_28_)^6−^ is a particular vanadate oligomer that is thermodynamically unstable at pH values above 6. However, it decomposes slowly having a half-life of about 9 h at pH 7.5 and 25 °C. This rate of decomposition increases substantially at higher pH values, for instance, at pH 12 and 25 °C, the decomposition of this compound is only about 1.5 h. In contrast, under acidic conditions, the stability of the compound changes significantly since the polyanion can suffer protonation. At pH around 1 and 25 °C, the half-life of decavanadate drops to about 6 s. At such strongly acidic conditions, the vanadate cation, VO_2_(H_2_O)_4_^+^, is the thermodynamically favorable species. The nature and concentration of the counterion also have a significant influence on the stability of the decavanadate polyanion along the pH range and the medium conditions, as indicated by the change of its decomposition rates in works of Soares et al. and Gândara et al. [134–137].

Vanadate can also interact with phosphates forming phosphovanadates such as H_n_VPO_7_^(4-*n*)−^, (*n* can be 1 or 2) at conditions of pH ≈ 7, where the pK_a_ of the compounds at an ionic strength of 0.15 M is 7.2 [138, 139]. This phosphovanadate compound is between one or two orders of magnitude less stable than divanadate against hydrolysis, but six orders of magnitude more stable than the diphosphate compound. Given the relatively high serum phosphate concentrations of 2.3 mM, phosphovanadates likely contribute to the physiological speciation of vanadium.

Additionally, the bone structure can act as storage for vanadate displaying a residence time of a month [77]. Other ligands, e.g., lactate (Lac), can promote the formation of coordination complexes with vanadium species, but this phenomenon is only favored under acidic conditions; at physiological pH formation of the complex is very unfavorable. Still, at slightly acidic conditions, the dominant lactatovanadium complexes are the di- and tri-nuclear bis(ligand) complexes of overall composition V_2_(Lac)_2_^2−^ and V_3_Lac_2_^3−^[140], where “V” stands for the oxide or dioxide vanadium center. At the physiological pH value (7.4), the VLac_2_ compound is the only existent species. Interestingly under acidic conditions, the mixed ligand system composed by vanadate, lactate, and citrate (Cit), forms a bi-nuclear complex of composition V_2_CitLac^*n*−^ (*n* = 2 or 3). Binary vanadate–citrate complexes in the physiological range of pH are restricted to a species of composition V_2_Cit^4−^ [77, 141].

## Vanadium Tissue Distribution and Cellular Uptake/Incorporation/Accumulation

Once in the bloodstream, vanadium is distributed and stored in different tissues. The contents of vanadium in plasma, decline in three phases: (i) The first phase is a rapid decline with a half-life of about 1 h, followed by (ii) a second intermediate phase where vanadium decline with a half-life ca 26 h, and moreover, (iii) a third slow phase where, on average, the half-life is approximately 10 days. Vanadium contents in blood are thus reduced to about 30% within the first 24 h, and about 50% is recovered in urine after 12 days [106, 115, 135]. Although the body clearance occurs directly via urinary excretion, while as long as the vanadium stays in the bloodstream, the distribution occurs towards different tissues such as the heart, liver, kidney, spleen, brain, muscle, adipose tissue, and bones. In this context, neutron activation analysis (NAA) has been one of the most important techniques used to determine the total vanadium levels in different organs.

### Vanadium Tissue Distribution from Humans and Animal Studies

The longer residence time of vanadium is in bones, where it replaces phosphorus in the mineral hydroxyapatite, Ca_5_(PO_4_)_3_OH, is over 1 month, which corresponds to a half-life of 4–5 days [18]. Analyses done utilizing NAA have found the following concentrations of vanadium in different human tissues (in ng/g of wet weight): fat and muscle, 0.55; heart, 1.1; kidney, 3.0; liver, 7.5; lung, 2.1; and thyroid, 3.1. [40, 142]. Also, studies have identified that the human colostrum and breast milk generally contained less than 1.0 ng/g of vanadium of dry weight [143]. Additionally, vanadium concentrations in scalp hair of healthy adults have been found in the range of 433 pg/g to 90 ng/g [144–147]. In general, the evidence has demonstrated that the most tissues contain less than 10 ng vanadium/g wet weight.

Studies with animals under a high vanadium content diet indicate a marked increase of the metal retention in various tissues. In rats, vanadium content in the liver increases from 10 to 55 ng/g of wet weight when the vanadium diet was increased from 0.1 to 25 μg/g [60]. Remarkably, the age of the animals represents a variable factor that needs to be considered. For instance, in rats between 21 and 115 days old, vanadium concentration decrease in the kidney, liver, lung, and spleen, but it exhibits an increment in fat and bone; however, variations in the brain, heart, testes, and spleen were small or negligible [148]. Parker and Sharma reported that rats continuously administered with drinking water ad libitum, containing 50 ppm of vanadyl sulfate and sodium orthovanadate during a 3-month period, showed increased levels of vanadium in the kidney, bone, liver, and muscle, and after 9 weeks of suspension of the vanadium administration, the concentration in tissues declined rapidly, except in bones [149]. Bone tissue is well established as one of the major body pool for vanadium retention [41, 58, 148, 150]. In this context, studies had found that in sheep bones, vanadium increased from 220 to 3320 ng/g of dry weight when dietary vanadium was increased from 10 to 220 μg/g [150].

Furthermore, studies in rats fed with VOSO_4_ showed the following trend in vanadium tissue concentrations: kidney > liver > bone > pancreas [151–153]. On the contrary, rats treated with different vanadium compounds such as VO(5-ipa)_2_, displays the following trend in vanadium tissue concentrations: bone > kidney > spleen > liver > pancreas > lung ≈ heart > blood cell ≈ serum > brain [154, 155]. Such differences in the organ distribution of vanadium between the animals fed with VOSO_4_ and those fed with other vanadium compounds suggest a distinct long-acting character of the different complexes. Along these lines, vanadium levels after 2-week administration of bis-maltolato oxovanadium (BMOV), 0.75 mg/mL in drinking water, were comparable with those obtained after 10 weeks of VOSO_4_ intake, albeit the daily dose administration of BMOV was about half of that of VOSO_4_, which vanadium accumulation was observed in the bone, kidneys, liver, muscle, and fat [156].

NAA and radioisotope determination studies found that for streptozotocin (STZ) rats treated with VO(6-Mepic)_2_, vanadium was accumulated in the majority of tissues with the following trend: kidneys > liver > bone > pancreas [157]. Also, EPR studies have shown that VO^2+^ species incorporated into the blood are first distributed to tissues that presented the short-time accumulation (liver and kidneys) and then to the tissues of long-time (bones) [140]. Real-time EPR analysis of VO^2+^ species revealed that the vanadium clearance rates from the bloodstream are distinct for different compounds. For instance, when rats are fed on the VOSO_4_ compound, the vanadium clearance rate is faster than when exposed to VO(5-ipa)_2_ or any other halogenated vanadyl complex, with a clearance rate of 5 min for VOSO_4_-treated rats and 7–30 min for other vanadium compound-treated rats. The difference in vanadium elimination rates from the circulating blood in rats indicates an important variation in the association between the vanadium compounds and the blood components such as serum proteins or erythrocytes [155].

### Cellular Mechanisms for Vanadium Cellular Incorporation

**Fig. 3:**
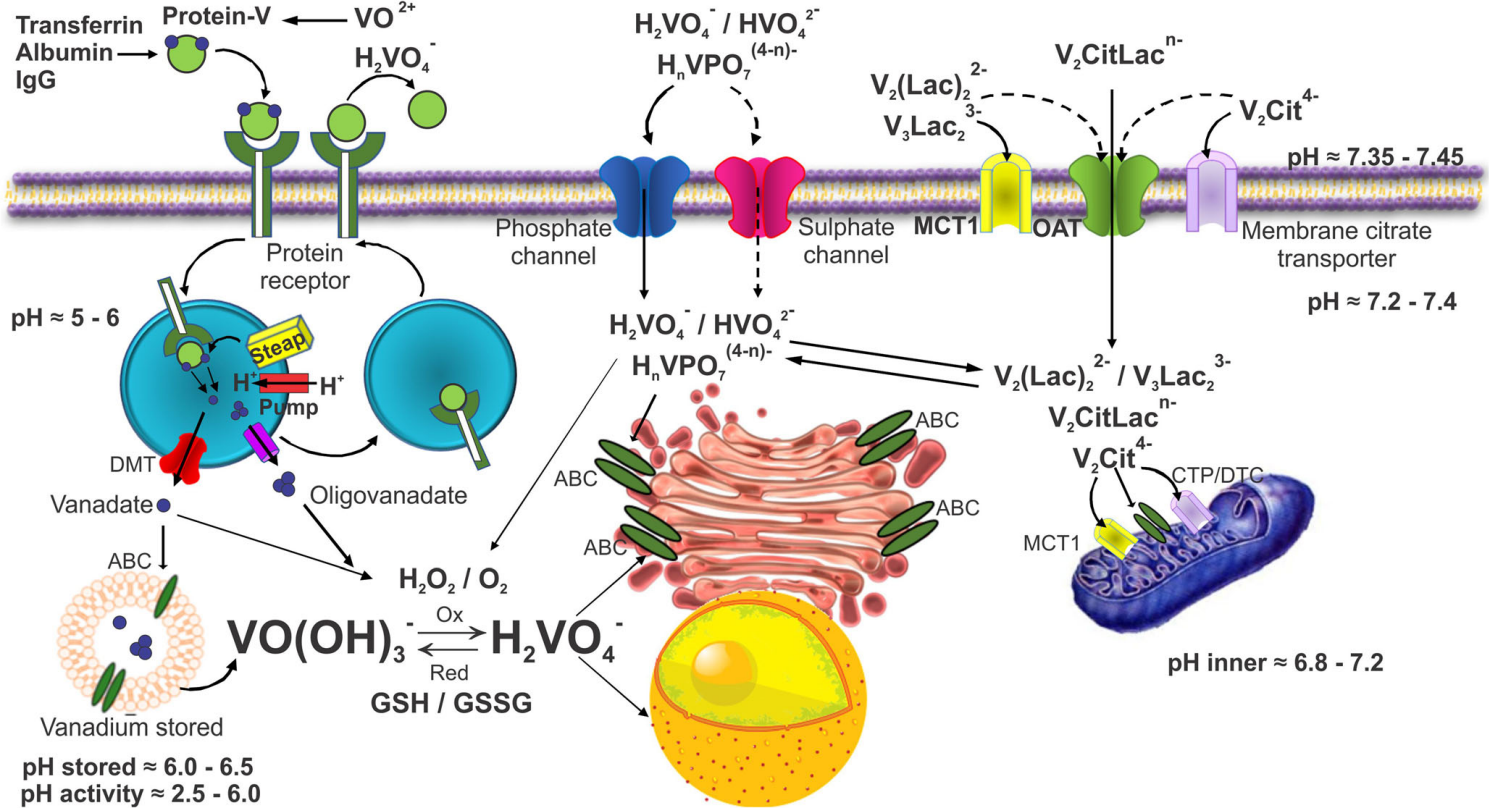
Vanadium species uptake and cellular compartmentalization. IgG, immunoglobulin; MCT1, monocarboxylate transporter-1; OAT, organic anion transporter, DMT1, divalent metal transporter 1; CTP, mitochondrial citrate transport protein; DCT, dicarboxylate-tricarboxylate carrier; ABC, ATP-binding cassette transporters; STEAP, STEAP metalloreductase

The *carrier* ligand (with the general equation VO(*carrier*)) largely influences the efficacy of a vanadium compound by determining its transport, stability, and bioavailability to different tissues. Particularly, the bioavailability of the vanadium compounds is of the utmost importance since it is linked to their therapeutic effectiveness [82]. Vanadium compounds reach cellular compartmentalization after the recognition process of the specific *carrier* ligand by a particular cell surface receptor occurs (e.g., transferrin, albumin, IgG) and subsequent endocytosis (Fig. 3). Then, proton pumps acidify the intra-vesicular environment and specific cellular events produced conformational changes that promote the vanadium release and cytoplasmic mobilization, which probably involves the divalent metal transporter-1 (DMT1) [158]. Once in the cytoplasm and depending on the pH and vanadium concentration conditions, oligovanadates may be formed. Additionally, vanadium species get into the cell by diffusion utilizing phosphate or sulfate channels, membrane citrate transporters, lactate transporter (monocarboxylate transporter, MCT1), and the organic anion transporter (OCT). After cellular uptake, vanadium compounds can be again subject to speciation and redox modifications, which will impact their subsequent bioavailability, site of interaction, and therapeutic or toxicity effects. Those effects will depend on several factors, such as the amount of the uptake, the type of body tissue, and the nature of the *carrier* ligand (if it is still present or not). In any case, the final intracellular breakdown of the complex most probably occurs to allow the display of vanadium’s physiological effects [108].

## Interconversion Between Vanadium Species and Cellular Redox Balance

The complexity of biological systems, coupled with the rich chemistry of vanadium in aqueous solutions, make the study of vanadium compounds in living systems very challenging. Within organisms, cells are divided into different organelles and vesicles by membranes, each compartment having different pH depending on the physiological, physiopathological, or pathological states, and different natural ligands hence different abilities to accumulate vanadium. Combining the effects of cellular architecture with the pH and concentration-dependent equilibria that govern vanadium chemistry can likely result in the formation of different oligomeric species with varying oxidation states, each found in different parts of the cell after an administration of a single vanadium compound. Also, vanadium and vanadium compounds inside the cells can interact with different proteins and act as inhibitor or activator (analog) and influence different signaling pathways. Several studies have demonstrated that vanadium can undergo speciation reactions in living cells. EPR and ^51^V NMR have provided evidence of the presence of vanadate tetramer formation and vanadyl species after the cells were exposed to the monomeric vanadate [159, 160]. ^51^V NMR also evidenced the formation of decavanadate (V_10_O_28_)^6−^ in cells at a pH of 6.5 and 5 mM vanadate concentration, showing that vanadium can be concentrated inside acidic cellular organelles [161]. It has been demonstrated that the distribution of vanadium inside the cell depends on the vanadium compound that is administered. The biodistribution of vanadium in fish red blood cells (RBCs), plasma and cardiac cytosol were found to depend upon the administration of either metavanadate or decavanadate [162]. Also, the ratio of vanadium in the plasma to vanadium in RBCs increased over time with metavanadate administration but remained constant for decavanadate administration [162, 163]. Although there is still some disagreement regarding the extent of the physiological importance of the decavanadate ion, multiple recent studies have conclusively demonstrated that decavanadate can induce changes in the biological activities of several enzymes, which underlines the importance decavanadate-based compounds in medicine [86, 87, 90–92, 134, 164–167].

Many of the beneficial or prejudicial physiological effects of vanadate are, at least in part, due to the structural and chemical similarities between orthovanadate and phosphate, H_*x*_PO_4_^(3-*x*)−^. However, a major difference between vanadium and phosphorous is the ease with which vanadium forms oligomeric metavanadate rings, such as [V_4_O_12_]^4−^, and polyoxovanadate clusters such as [V_10_O_28_]^6−^. Another significant difference is the ability of V^+5^ to get reduced to V^+4^ in the form of vanadyl in vivo by thiol-containing species such as cysteine and glutathione. Indeed, various forms of vanadium that exert different biological functions undergo biotransformations [62, 68, 70, 168, 169]. Undoubtedly, the degree to which pentavalent V^5+^ is reduced to tetravalent V^4+^ is an important factor influencing how much metal/compound is transported into/out of cells, the magnitude of reactions involving the superoxide anion (·O^2−^) and hydrogen peroxide (H_2_O_2_), and the key cellular processes that are potentially impacted by those changes [170–172].

**Fig. 4:**
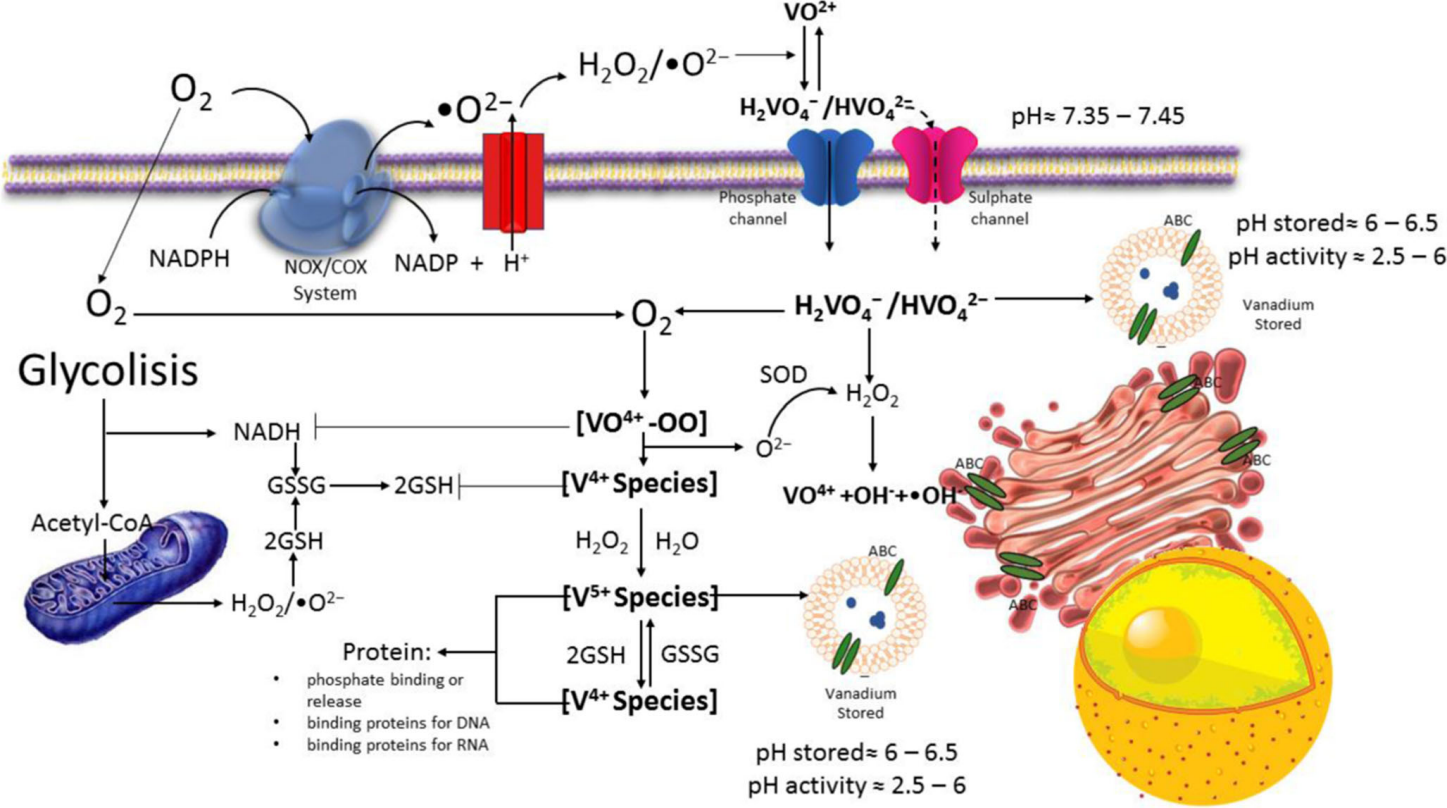
Vanadium interconversion species, redox balance, and oxidative stress. NADPH, reduced form of nicotinamide adenine dinucleotide phosphate; NADP, the oxidized form of nicotinamide adenine dinucleotide phosphate; NADH, the reduced form of nicotinamide adenine dinucleotide; SOD, superoxide dismutase; GSH, the reduced form of glutathione; GSSG, the oxidized form of glutathione; NOX/COX, NADPH oxidase system

Interconversion between vanadium species (mostly V^+4^/V^+5^ and in less degree in V^+3^) is constantly occurring inside of cells. Previous studies strongly suggest that vanadium-ligand complex is not stable in the body. Thus, the vanadium administered will seek the speciation required for an equilibrated distribution. This behavior proves that vanadium complexation and speciation is a dynamic process in an environmental hydraulically unstable (Fig. 4). The ligands available for complexation with the dissociated vanadium will be determined by the cellular compartment or body fluid in which the dissociation occurs. Therefore, understanding the oxidation-reduction interactions of vanadium is important to understand the effects of therapeutically. The natural cellular reducing compounds glutathione (GSH) and ascorbic acid interact and readily reduce vanadium from V^+5^ to V^+4^. In oxygen-depleted regions, reduction will be complete but in the presence of oxygen, a redox equilibrium will be established [173]. The GSH system is part of the thiol cycle in mammalian cells that may transduce oxidative stress redox signaling into the induction of many genes involved in proliferation, differentiation, and apoptosis [174]. Although GSH is a rather ineffectual reducing agent, redox interactions stabilize the oxidation state of vanadium through the complexation with oxidized GSH (GSSG). A high intracellular excess of GSH increases the possibility of VO^2+^ formation and its complexation with either GSH or GSSG. Both GSH and GSSG have been shown to be reasonably potent binders of VO^+2^ [129, 175–177]. Other effective reducing agents, such as NAD^+^/NADH, NADP^+^/NADPH, FAD^+^/FADH, or ascorbate, may interconvert V^5+^ and V^4+^, as well as V^3+^ species [18, 178–180].

Reactive oxygen and hydrolytic degradation of VO^2+^ may be responsible for the reoxidation to vanadate. The redox potential for the H_2_VO_4_^−^/VO^+2^ pair at pH 7 is − 0.34 V, which is comparable to − 0.32 V for the NAD^+^/NADH. Vanadyl has also been shown to stimulate NADH oxidation by a rapid phase that involves the production of vanadate followed by the production of H_2_O_2_ and (·O^2−^) [161, 181–184]. EPR studies have implied that the vanadate-mediated hydroxyl radical generation from superoxide in the presence of NADH was due to a Fenton mechanism rather than a Haber-Weiss reaction [185].

The chemical mechanism of the reaction of NADPH oxidation is a consequence of vanadate stimulation that generates a free radical chain system, in which increases of O_2_^−^ are generated [186, 187]. Decavanadate has been shown to be a more potent stimulator of the vanadate-dependent NADH oxidation activity than orthovanadate [188, 189]. The reductase activity of decavanadate is linked to the alternative activity of an NADP specific isocitrate dehydrogenase [190]. The role of these interesting plasma membrane-dependent, vanadate-stimulated NADPH oxidation reactions in cellular metabolism remains to be elucidated, although multiple interactions with components of the cellular metabolism are possible including interactions with xanthine oxidase and lipid peroxidation [191]. Decavanadate has been shown to enhance cytochrome c reduction [189], and cytochrome c release from mitochondria is associated with initiation of apoptosis [192]. Although, it has shown a dependence on the concentration, cellular speciation, as well as antioxidant defense level or even of other cellular protection systems.

The aqueous chemistry of vanadium allows vanadium to participate in cellular redox reactions involving both reactive species of oxygen and nitrogen (ROS and RNS). In different systems, vanadium stimulates nitric oxide formation or inhibits the stimulation of nitric oxide by cellular effectors. The final effect of increases of ROS and RNS on cell membranes that are very sensitive to oxidation is lipoperoxidation [192]. Lipid peroxidation reactions correlated with a decrease in the V^4+^/V^5+^ redox potential and proceeded without formation of radicals. Vanadium compounds can form a vanadium superoxide complex that acts as an active oxidizing species or decomposes to form hydroxyl radicals, which are known initiators of lipid peroxidation. Acute and chronic exposure to vanadium compounds causes oxidation of fatty acid lipids in both human erythrocytes and animals [142, 193]. In leukocytes, vanadium correlates with the formation of the ROS and depends on the activity of calcium channels [194]. Neutrophils activated with (V^4+^) vanadium species showed increased hydroxyl radical formation capacities and attenuation of myeloperoxidase activity, whereas the species with oxidation state + 5 did not show these effects [195]. There is evidence that supports a linking to vanadium and the nitric oxide signaling. Formation of radicals after addition of vanadyl sulfate to isolated perfused lungs induced constriction of pulmonary arteries accompanied by increased amounts of NO via protein kinase C [196].

## Vanadium and Intracellular Proteins Interaction

Vanadium displays high affinity for iron-containing proteins and, hence, a direct interaction with the intracellular protein ferritin, which has a high capacity for storing iron, has been suggested. In this context, vanadium is found naturally in horse spleen ferritin at levels of 5 to 10 vanadium atoms per protein, and interestingly, it exhibits a pH dependence with decreasing VO^2+^/protein ratios as the pH increases (e.g., 61%, 36%, and 27% at pH 6, 7, and 8, respectively), using a ratio VO^2+^/protein of 16 [88, 93, 197]. By using EPR spectroscopy, V^+5^ and V^4+^ species were detected in rats fed under vanadium-rich diets, particularly in the ferritin proteins from the liver, kidney, and spleen [113, 115, 198]. The EPR experiments indicate that the signals arise from the complex formed in the ferritin’s interior between iron and the VO^2+^ species. Moreover and due to the interaction above, VO^2+^ has been used as a spin probe to identify the binding sites not only for the natural substrates Fe^2+^ and Fe^3+^ but also to characterize the iron deposition inhibitors, Zn^2+^ and Tb^3+^. During the formation of the VO^2+^–apoferritin complexes, an average stoichiometry of 0.5–0.6 VO^2+^/subunit is observed, which corresponds to 12–16 VO^2+^ ions bound per 24-subunit protein. Metal ion hydrolysis decreases the concentration of the VO^2+^–apoferritin complex when the pH ranges from 6.0 to 7.0. While VO^2+^ binding to the specific metal sites of other metalloproteins suppressed the hydrolysis of vanadium, apoferritin is unique in allowing the hydrolysis process to occur; the analogous reaction with Fe^3+^ is a requirement of the formation of the iron core. Regarding its susceptibility to hydrolysis and its EPR properties, the VO^2+^–apoferritin complex behaves similarly to VO^2+^ complexes [199, 200].

On the other hand, ATPases are enzymes that catalyze the hydrolysis of phosphate–anhydride bonds with many important roles in biology namely in cellular energy metabolism. A wide range of affinities for vanadate are observed depending on the type of ATPases [87, 168, 201, 202]. The inhibitory effect of vanadate on some ATPases may vary from those corresponding to nM inhibition constants for the Na^+^, K^+^-ATPases [203]. The Na^+^-K^+^-ATPase is very tightly inhibited by vanadate with an association constant of 2.4 × 10^8^ M^−1^ [203, 204]. The inhibitory effect of vanadium also has been observed in ion pumps such as the H^+^/K^+^-ATPase or Ca^2+^-ATPase [168, 205–207]. Interestingly, decavanadate [V_10_O_28_]^6−^ is a more potent Ca^2+^ATPase inhibitor than monomeric vanadate [207–209]. The oxidation of a cysteine residue through reduction of the vanadate apparently is the inhibition mechanism of decavanadate to Ca^2+^-ATPase [205–209]. Myosin is considered as an ATPase because contains a motor domain comprising two binding sites responsible for interacting with the actin and ATP hydrolysis (the head); meanwhile, the intermediate domain arm increases the conformational change caused by ATP hydrolysis and is responsible for the binding of regulatory light chains like calmodulin. The tail contains a coiled coil and a targeting domain contributing to the enzyme specificity [210, 211]. Experiments of myosin inhibition have been demonstrated with vanadate [210]. Myosin type II binds vanadium in Ser236, a critical residue for the protein activity [211]. Vanadium also is related to myosin type I and type IV [212]. Monomeric vanadate mimics the transition state for the phosphate hydrolysis [213], blocking myosin by the ADP–phosphate intermediate state. Decavanadate also inhibits myosin ATPase and Ca^2+^-ATPase [205–208]. Decavanadate induces the formation of the intermediate myosin–MgATP–V10 complex blocking the contractile cycle, most probably in the pre-hydrolysis state [211]. In fact, by blocking the Ca^2+^ release, the contraction of the calcium pump and/or the actomyosin release of the metabolites prevents the relaxation of the muscle [211, 212].

### Vanadate and Phosphate

Most of the investigation done so far with vanadium and metalloproteins is directed towards the exploitation of the similarity between the phosphate and vanadate groups. The inhibition and stimulation of phosphate-metabolizing enzymes are commonly, and convincingly, traced back to what is termed “the vanadate–phosphate antagonism,” due to the similar physiological behavior of the two anions. In general terms, the vanadate and phosphate groups are indeed very similar to each other: with a tetrahedral morphology and almost-spherical outer-sphere charge distribution. The net ionic charge of the main species present at pH 7 is, however, different, − 2 in the case of phosphate and − 1 in the vanadate, and this can result in distinct interactions with electrophilic groups. There are other important differences, which are, at least in part, responsible for the inhibitory effect of vanadate towards phosphate-metabolizing enzymes. The main differences lie in the susceptibility of vanadate to (one-electron) reduction as a consequence of the presence of energetically low-lying *d* orbitals and coordination numbers larger than 4, usually 5 and 6. This has a consequence of generating five- or six-coordinated anions, the fixation of vanadate by coordination to functional groups provided by amino acid side chains of the proteins [214, 215]. Structurally, vanadate can be a competitor in sites commonly occupied by phosphate [216]. However, due to the different pK_a_, at physiological pH and ionic strengths, vanadate is mostly present as either H_2_VO_4_^−^ or HVO_4_^2−^, depending on the pH (6.8–7.4), while phosphate favors the HPO_4_^2−^ and H_2_PO^4−^ forms that exist in approximately equal amounts at the same pH range.

Kinases and phosphatases are enzymes that perform the addition or removal of a phosphate group, respectively. These enzymes modulate intracellular signaling pathways triggering a cascade of different physiological effects. In biological systems, the level of phosphorylation in proteins works as a balance resulting from the action of kinases and phosphatases. Thus, both types of enzymes have important roles in the regulation of cellular processes.

### Phosphatases

Phosphatases catalyze the hydrolysis of phosphate esters and can be classified into two groups: serine-threonine phosphate proteins (PSPases) and tyrosine-phosphate proteins (PTPases), depending on the identity of the amino acid residue in the catalytic site. The alkaline phosphatases, which have a serine residue in the active site, hydrolyze phosphate monoesters groups from small molecules and proteins and catalyze the transfer of phosphate to hydroxyl groups of organic molecules. In acid phosphatases, a histidine residue at the active site is phosphorylated by the substrate and the phosphate group also catalyzes the reaction. In both types, the reaction mechanism of hydrolysis carried out by phosphatases involves the formation of a 5-coordinate high-energy transition state. These enzymes are inhibited by vanadate, which is often considered to act as a transition state analog (TSA) of phosphatase-catalyzed reactions [217–220]. Vanadate is not a specific inhibitor of all phosphatases but can be a potent inhibitor of the activity, because it can mimic the 5-coordinate transition state of phosphate formed during the phosphatase catalytic cycle. Vanadate can also cause cysteine oxidation at the active site, thus, affecting the function of several PTPases that require thiol-reducing agents for optimal activity [221–224]. Likewise, it is accepted that during the dephosphorylation process, the cysteine of the active site is in the thiolate state (RS^-^) and very susceptible to oxidation. This phenomenon can occur by reaction with neighboring peptide backbone atoms, inducing important conformational changes in the active site. In both cases, the outcome is the inactivation of the enzyme. The vanadate anion also shows a trigonal bipyramidal geometry, coordinated by His in the apical position, previously implicated in the hydrolysis with hydrogen bonds between vanadate O-atoms and His-Asp (257 and 258 position, respectively) suggesting that the former is involved in the stabilization of the negatively charged transition state intermediate while the latter is assuring protonation of the substrate during reaction mechanism. V^5+^ compounds form vanadate complexes with both acid and alkaline phosphatases and much of them act as enzymatic inhibitors [71, 225–228].

The PTPase proteins can dephosphorylate a large variety of tyrosine–phosphoryl bonds, independently of the overall structure of the substrate protein, so vanadate has been widely used to study the reaction mechanism of these enzymes. Even though some authors claim that vanadate is not a true substrate analog [229, 230], several crystal structures of V^5+^–PTP complexes have been determined to provide important information regarding transition state conformations and structural determinants for catalysis [230–234]. Particularly, the protein tyrosine phosphatese 1B (PTP1B), a key enzyme in the insulin signaling pathway, possesses two relevant TSA, a tyrosine site where vanadate is bound similarly to the active site as the phosphorylated tyrosine substrates (i.e., adopting a trigonal bipyramidal geometry with the nucleophilic cysteine and the tyrosyl oxygen in apical positions) [235] and a Cys-bound vanadate [230]. Similar to the phosphoenzyme before the inorganic phosphate release [236, 237], the oxoanion exhibits a double distorted trigonal bipyramid containing a cyclic [VO]_2_ core [231]. However, as recently reported, vanadium bound to phosphatases also shows a square pyramidal geometry [238]. The application of X-ray crystallographic data to map out the structures and geometries in the phosphatase active site along the energy surface of the phosphate ester hydrolysis has been described. Hengge et al. collected the available structures to investigate the reaction pathway for PTP1B using the CShM method [231]. Briefly, the enzyme begins the catalytic cycle as a free cysteine in the protein [239]. After binding the substrate analog, in the form of a phosphorylated amino acid Tyr, a Michaelis complex containing an unusually long bond between the cysteine and the vanadate is formed [231]. This corresponds to the first 5-coordinate transition state on the pathway where vanadium adopts a trigonal bipyramidal geometry. Some computational results suggest that in the transition state of the V–PTP1B complex, the V–S, and V–O bonds adopt the 2.5 Å and 2.1 Å bond lengths, respectively [238]. The fact that vanadium forms protein complexes with these unusual bond distances supports the possibility that the transition states may also present distortions to the umbrella conformations that are particularly favorable to catalyzing the monoesters hydrolysis [240]. This may be related to the mechanism of phosphatases, where the explored transition states are more prevalent than in enzymes catalyzing phosphotriester or diester hydrolysis [241]. Inhibition of PTP1B by vanadate has been demonstrated in vitro and in vivo studies and substantiated by using single-crystal X-ray diffraction and two-dimensional ^1^H-^15^N NMR spectroscopic techniques. The compounds that are mostly used for these inhibitory studies are vanadyl sulfate or bis(maltolato)oxovanadium (BMOV) [241]. Irrespective of the nature of the administered vanadium species, the same compound with incorporated vanadate was obtained, which adequately demonstrates that the active species is the vanadate ion, formed by the elimination of the ligands and the oxidation of V^4+^ to V^5+^. Likewise, EPR studies made evident the reaction between VO^2+^ and the residues at the PTP1B active site that coordinate the vanadyl ion without a coupled redox interaction. The preferred coordination site of vanadium depends on the pH. Thus, the preferential phosphatase binding site in an acid environment is via a histidine residue, whereas in alkaline conditions, the coordination occurs via a cysteine residue [240, 241].

The inhibition or activation of some phosphatases occurs by the formation of analog compounds to phosphate esters as in the case of vanadate esters. Vanadate esters are readily formed in aqueous solutions. However, they are not particularly stable species in these conditions since they are not only readily hydrolyzed but also displayed formation constants in the order of 10^−1^–1 M^−1^. The position of these equilibria depends on vanadate concentration, ionic strength, and pH.

### Kinases

Another important enzyme group where the effects of vanadium have been extensively investigated are kinases, which are responsible for the transfer of a phosphate group. In 1995, Arvai and coworkers reported the complex formation of vanadate and a human regulatory subunit of the cyclin-dependent kinases (CDK) [242, 243]. Subsequent studies indicated that vanadium compounds could also indirectly inhibit the activity of CDK2 in cyclin-A and cyclin-B complexes inducing G2/M phase arrest [244]. Mitogen-activated protein kinases (MAPK), mainly ERK and p38, are also activated by vanadium compounds resulting in G2/M phase arrest [245, 246]. Additionally, vanadate treatment triggers the phosphorylation of the retinoblastoma protein (pRb) and the release of the transcription factor E2F1 that is a component of the downstream proliferative machinery regulated by protein kinase B (PKB also known as Akt), which impacts cell growth, survival, and metabolism. Furthermore, vanadate increases Akt kinase activity and causes its phosphorylation at Ser473 and Thr308, consequently increasing the number of cells at the synthesis (S) phase and transition from gap 1 (G_1_) to S phase through the E2F-pRb pathway in normal C141 cells [247]. Other studies show that vanadium compounds stimulate kinases in the signal transduction pathways used by insulin beyond the insulin receptor (IR) and the substrate IRS-1, and secondarily, the phosphoinosidide 3 kinase (PI3K), Akt, MAPK pathways (mainly, ERK pathways) together with the activation of the S6 kinases, hence playing an anti-diabetic and anti-lipolytic role, with concomitant insulin-like effects [248–256]. Vanadate also stimulates the IRS-1 phosphorylation, the PI3K activity, the ERK signaling pathway, and the p70s6k and p90rsk kinases independently of IR-tyrosine phosphorylation, which in turn phosphorylate and regulate the activity of several transcription factors related with cell proliferation and glycogen synthesis [257–259]. Moreover, the activation of the ras-MAP kinase signaling pathways by the VOSO_4_ compound seems to depend on the activity of PI3K [257, 260]. Vanadate may also cause some of the insulin-like effects through the activation of a cytosolic kinase (CytPTK) that stimulates lipogenesis and glucose oxidation (via glycolysis and the pentose phosphate pathway) [253, 254]. The oxidation from V^4+^ to V^5+^ promotes the generation of the pervanadate compound as an intermediate that triggers glucose uptake by increasing autophosphorylation of the IR to prevent its dephosphorylation. The pervanadate species also acts as insulin enhancer, because it has the unique ability to markedly increase the maximal cell responsiveness in the stimulation of the glucose transport achieved at a saturating insulin concentration [261–263].

## Vanadium Signal Transduction Cascades and Therapeutic Implications

The metabolic disorders of lipids and carbohydrates are strongly linked to obesity development, insulin resistance, type 2 diabetes mellitus (T2DM), dyslipidemia, hepatic steatosis, and cardiovascular disease. All these complications contribute to the puzzle called metabolic syndrome, which is a series of conditions that when occurring together, increase the risk of heart disease, stroke, and diabetes. Metabolic syndrome is a major problem of public health and an important clinical challenge worldwide. The International Diabetes Federation estimates that one-quarter of the world’s adult population has metabolic syndrome associated with overweight and a high body mass index that it is reflected in a higher body fat mass, mainly distributed in the visceral adipose tissue [264–267].

### Vanadium Anti-diabetic Compounds in Animal and Human Models

In this regard, vanadium compounds have emerged as possible therapeutic alternatives to the current treatment of diabetes. The first clinical trials using simple inorganic vanadium compounds to treat diabetic individuals were performed in the 1990s [268–270]. Subsequently, studies done to test the therapeutic properties of more complex vanadium compounds such as bis(2-ethyl-3-hydroxy-4-pyronato)oxovanadium(IV) (BEOV) and bis(3-hydroxy-2-methyl-4-pyronato)oxovanadium(IV) (BMOV) in streptozotocin-diabetic rats showed interesting anti-diabetic benefits and improved efficacy when compared to common inorganic vanadium salt such as vanadyl sulfate [271–274]. Over time, BMOV has become the benchmark compound against which many vanadium-based hypoglycemic agents have been compared [271, 275]. Although very promising, diabetes-related studies of vanadium have some issues. For instance, since BEOV and BMOV were described, the literature frequently refers to vanadium compounds as insulin-mimetics. However, the majority of the studies have only shown hypoglycemic or hypolipemic effects. Moreover, diabetic models are mainly (STZ) induced, which are not always able to attain complete insulin depletion, making the vanadium therapeutic efficacy dependent on the severity of hyperglycemia and residual insulin in the pancreas. Also, the STZ-induced model causes not only beta cell necrosis but also DNA alkylation in different tissues. Furthermore, this murine model causes severe cytotoxic effects related with the transport capacity of glucose through the glucose transporters GLUT-2 and GLUT-1 in the pancreas, liver, kidney, and brain, producing tissue-specific cell death and structural and metabolic changes. All these issues complicate the correct anti-diabetic evaluation of vanadium compounds using this model [276–279]. The hypoglycemic potential of various vanadium compounds has also been examined in models that present features similar to those observed in types 1 and 2 diabetes mellitus. In models that resemble type 1 diabetes, administration of polyoxovanadates (e.g., decavanadate) improves serum glucose levels and glucose tolerance, albeit the insulin deficiency improvement has been demonstrated in a limited number of studies [92, 280–286]. Indeed, decavanadate has demonstrated to be more efficient than BMOV in inducing glucose uptake in rat adipocytes [287]. Vanadium therapy has also been investigated in diabetic humans. In type 1 diabetes patients, oral administration of sodium metavanadate and vanadyl sulfate in doses of 50–125 mg/day during 2 to 4 weeks, improves fasting plasma glucose levels and daily insulin requirements in type 1 diabetic patients. Similar doses administrated to type 2 diabetes subjects showed an increase in insulin sensitivity, reduction in fasting plasma glucose levels and glycosylated hemoglobin (HbA_1c_), and alleviation insulin resistance, besides suppressing endogenous hepatic glucose production [45, 268, 269, 288–291].

### Physiological Determinants of Vanadium Anti-diabetic Action/Effects

#### Vanadium and Type 1 Diabetes Mellitus

Remarkably, important elements in the vanadium mechanism associated with glucose regulation are the glycogen synthesis recovery, the uptake enhancement, and the utilization of glucose; type 1 diabetes models treated with vanadium have shown glycogen increase in muscle and heart, which suggest an improvement in the insulin signaling pathway associated with the reestablishment of the GLUT-4 expression; however, the therapeutic dose must be finely managed [292, 293]. Studies in STZ-induced diabetic rats treated orally with either vanadium coordination compounds (i.e., III-, IV-, and V-chlorodipicolinate (Vdipic-Cl)) or inorganic vanadium salts (i.e., vanadyl sulfate or sodium metavanadate) via drinking water during 28 days showed significantly improved hyperglycemia and glucose intolerance. The animals also showed increased hepatic glycogen synthesis and restored mRNA levels of glycolytic enzymes in the liver such as phosphoenolpyruvate carboxykinase (PEPCK), glucokinase (GK), and L-pyruvate kinase (L-PK), which are frequently altered in diabetic animals. While both types of vanadium salts and compounds elicited anti-diabetic effects, the best results were observed in rats administered with Vdipic-Cl [281].

#### Vanadium and Carbohydrates Homeostasis in Type 2 Diabetes Mellitus

In type 2 diabetes models (e.g., *db*/*db* mice, sucrose-fed rats, fa/fa Zucker rats), the administration of vanadium salts and vanadium compounds normalizes the glycogen synthase activity, while in the non-diabetic controls, no alterations in the enzymatic activities were observed [294–297]. On the other hand, in genetically modified mice (*ob*/*ob*), hepatic glycogen and glycogen synthase activity was not restored, despite the normalization of serum glucose level, which strongly suggests that vanadium treatment in this particular model favors the de novo lipogenesis because of an increase in body weight [298]. In this context, BMOV treatment during 7 weeks in STZ-diabetic rats failed to improve insulin-stimulated glycogen synthase activation in the skeletal muscle [299], whereas similar treatment enhanced it in fa/fa Zucker rats [300]. Treatment during 4 and 8 weeks with the metformin-decavanadate compound in alloxan-diabetic rats did not show significant amelioration in the glycogen levels, while similar treatment in diabetic rats induced by a hypercaloric diet displayed improvement in glycogen concentration in the liver, muscle, and renal cortex (but not in the heart and renal medulla) [91, 92]. Cell culture experiments using mouse diaphragm, rat hepatocytes, rat diaphragm, rat adipocytes, Chinese hamster ovary cells overexpressing insulin receptor (CHO-HIR), and 3T3-L1 adipocytes showed that the addition of vanadium salts and compounds also enhances the glycogen synthesis [17, 249–251, 258, 301–304]. In humans, vanadyl sulfate treatment (150 mg/day for 6 weeks) caused a 1.5-fold enhancement of glycogen synthase fractional velocity but failed to alter either basal or insulin-stimulated glycogen synthase activity, which suggests that vanadium could activate the kinases involved in the glycogen synthesis without the necessity of insulin stimulus [45]. In addition to the stimulatory action on glucose uptake and utilization, vanadium-induced suppression of hepatic glucose output also improves glucose homeostasis. Vanadium treatment decreases the over-expression PEPCK and glucose-6-phosphatase (G6Pase) main gluconeogenic enzymes [305–311].

Therefore, in both diabetic animals and humans, the administration of vanadium decreases the hepatic glucose production [284, 285, 312], although discrepancies in this respect still exist [45, 268, 269, 288, 289]. Finally, the direct biochemical control of glucose homeostasis for vanadium treatments is associated with the enhancement in glycolysis and glucose oxidation as observed in isolated rat adipose tissue and hepatocytes HepG2 cells [17, 281, 301, 313]. These effects are attributed to selective stimulation of the pentose phosphate pathway and concomitant production of fructose-2,6-bisphosphate (Fru-2,6-P2), the main regulatory metabolite of this pathway.

The effect of vanadate on the Fru-2,6-P2 levels displayed a time and dose dependency [314]. Likewise, vanadate does not modify the 6-phosphofructo-2-kinase and pyruvate kinase activities, and it does counteract the inactivation of these enzymes induced by glucagon. Lastly, vanadate can increase the production of both lactate and CO_2_ in hepatocytes from STZ-induced diabetic rats; hence, behaving as a glycolytic effector in these cells, this effect may be related to its ability to normalize blood glucose levels in diabetic animals [315].

#### Vanadium and Lipids Homeostasis in Type 2 Diabetes Mellitus

The *novo* lipogenesis is a natural pathway to control of glucose levels, encouraging the triglycerides biosynthesis in the liver, which is dependent on a correct insulin signaling on lipogenic pathways. However, in insulin resistance, obesity, dyslipidemia state, and diabetes mellitus, the hypertriglyceridemia and breaking of the balance between lipogenesis and lipolysis have been observed as a common factor. Lipolysis in isolated adipose tissue was normalized in vanadyl-treated diabetic animals [316, 317]. On the other way, in genetically modified type 2 diabetes models, vanadium has demonstrated inconsistent results, probably linked to the genetic background. In Zucker fa/fa rats with vanadium treatment, the FFA levels did not change [318]. In a systematic investigation of the anti-diabetic properties and anti-lipolytic effect of non-oxide V^4+^ complexes, results showed that vanadium compounds did not cause any inhibition of free fatty acid (FFA) fluxes [81]. Along these lines, the water-soluble 3-hydroxy-4-pyridinonato oxidovanadium(IV) complexes proved to be insufficient to inhibit FFA release; however, the complex bis(3-hydroxy-1(H)-2-methyl-4-pyridonato)oxidovanadium(IV) was able to inhibit FFA release to a larger extent than vanadyl sulfate [319]. Bis(allixinate)oxovanadium(IV) which contains allixin, a garlic component, has demonstrated a high in vitro insulin-mimetic activity regarding FFA release in isolated adipocytes from type 1 diabetic mouse model, after both intraperitoneal injections and oral administrations [320]. Yet, the decavanadate administration has shown the best lipid regulation. Previous studies showed that hexaquis(benzylammonium) decavanadate ((C_7_H_10_N)_6_[V_10_O_28_]·2H_2_O or B6V10 for short), a conjugate salt of benzylamine and decavanadate, can normalize the plasma concentration of non-esterified fatty acids after a chronic administration in severe diabetes rat or mouse models [17]. Moreover, the putative anti-lipolytic actions of B6V10 in murine and human adipocytes tested with increasing doses of 0.1 to 100 μmol/L on the triglyceride breakdown (lipolysis releasing of FFA and glycerol) demonstrated its efficient anti-lipolytic activity. Lipid-lowering and metabolic regulation activity of metforminium decavanadate (H_2_Metf)_3_[V_10_O_28_]·8H_2_O (MetfDeca) was also observed in insulin-requiring and non-insulin-requiring animal models [91, 92]. Lipid metabolism behavior suggested an improvement in tissues, specifically about energy-obtaining mode because the rates of hepatic triglyceride synthesis from fatty acid esterification are dependent on substrate flux and independent of the circulating plasma insulin concentrations. Thus, when serum FFA diminishes liver lost flux of prime matter to build triglycerides, results strongly suggest that MetfDeca induced lipidic burning, as in T2DM model [321, 322]. Furthermore, sodium metavanadate and vanadyl sulfate decreased plasma cholesterol levels in humans without alteration of either plasma free fatty acid or triglyceride fractions [45, 268, 288, 289]. Vanadate has also been shown to reduce total and free cholesterol levels in normal subjects, which may be due to inhibition of the steps involved in cholesterol biosynthesis [323, 324]. In isolated hepatocytes [325] and adipocytes [158], sodium metavanadate modulated lipid metabolism by stimulating lipogenesis and suppressing lipolytic activity.

#### Vanadium and Insulin Signaling in Diabetes Mellitus

**Fig. 5:**
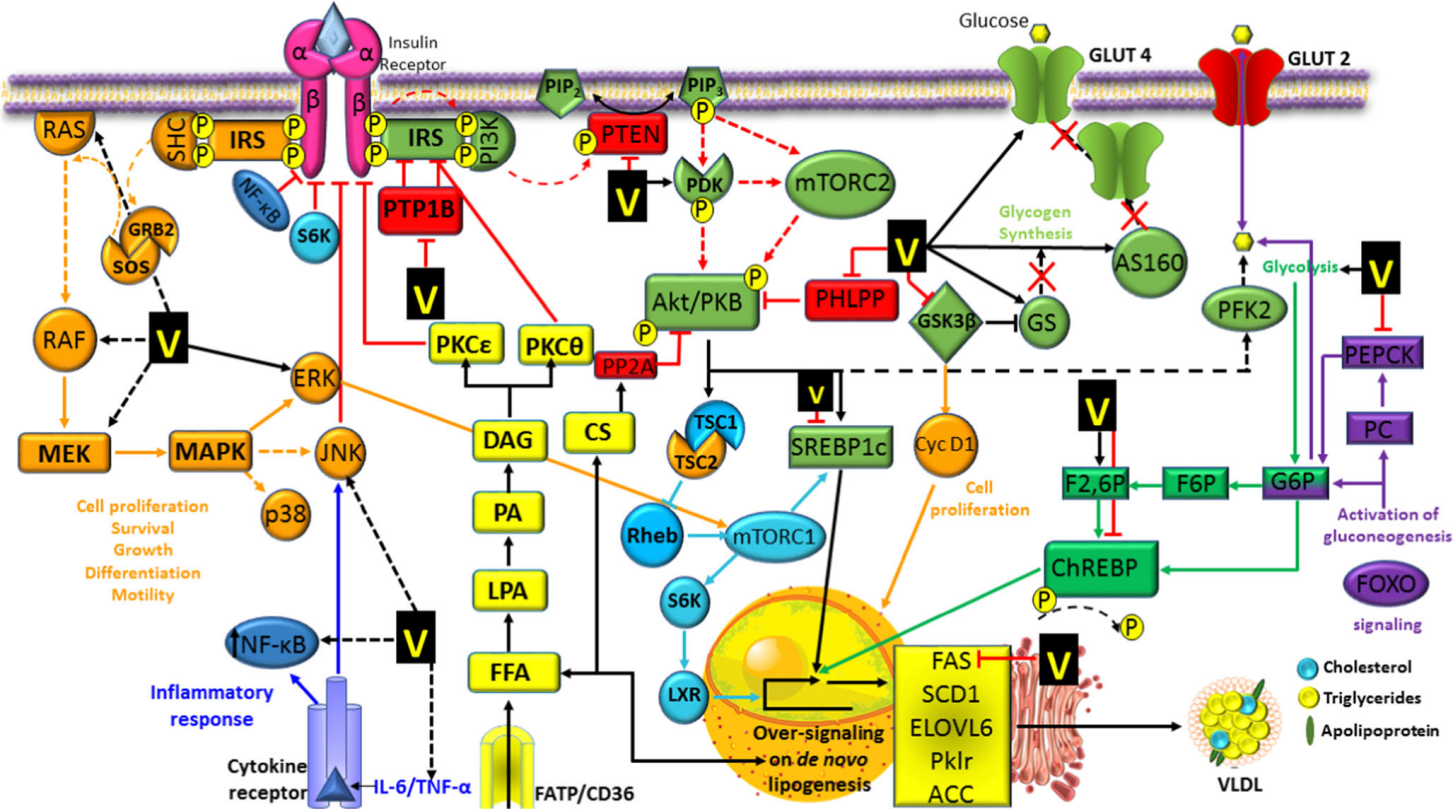
Insulin resistance mechanism and potential sites of vanadium activity. Red dashed arrows indicate changes in phosphorylation sequence of the insulin signaling cascade. Red solid lines imply inhibition of the signaling or actions. Red crosses indicate loss of action. Purple boxes represent gluconeogenesis activation. Yellow boxes depict the free fatty acid uptake. Blue boxes indicate over-stimulation of SREBP1c. Orange boxes represent the MAPK pathway. Red boxes are critical phosphatases. Blue boxes show inflammation pathways. Black boxes with yellow V are critical points or potential sites of vanadium activity

One of the most studied signaling cascades linked to vanadium compounds is the insulin-activated pathway associated with phosphatase inhibition, mainly PTPases. In healthy subjects, the receptor and specific insulin-response substrates are phosphorylated after insulin binding, but in diabetes mellitus (type 1 or type 2), there is an insufficient or anomalous response of the cellular insulin receptors to the hormone and therefore to the signal transduction cascades. At the molecular level, most of the effects observed in the presence of vanadium occur through IRS-1 phosphorylation due to the potent PTPases inhibitory properties of vanadium salts (Fig. 5) [229–234, 326]. A major intracellular target for vanadium is the PTP1B, which regulates the phosphorylation process between the insulin receptor and its substrate IRS. Inhibition of PTP1B activity allows the insulin receptor (IR) to remain activated, that is, to retain the tyrosine phosphorylation of the IR-β subunit [278, 327, 328]. Therefore, it has been suggested that by preventing dephosphorylation of the IR-β subunit, vanadium may ameliorate the activity of IR protein tyrosine kinase (PTK).

Vanadium treatment has also been proposed as a modulator of mitogen-activated protein kinases (MAPK) pathways (see Fig. 5). Pandey and coworkers demonstrated that vanadyl sulfate treatment resulted in an increased level of tyrosine phosphorylation of ERK 1/2, stimulation of MAPK kinase (MEK) and C-raf-1 activities, and activation of p21ras and ribosomal protein 6 kinase (S6K). Also, wortmannin and LY294002, two structurally and mechanistically different inhibitors of PI3K, can block the vanadyl sulfate-mediated increase in MAPK activity and phosphorylation of ERK 1/2 and S6K. These results suggested that the vanadyl sulfate mechanisms are mediated by the PI3K-dependent stimulation of the ras-MAPK and S6K pathways [258, 329, 330]. Isolated rat adipocytes treated with 1 mM sodium vanadate displayed a rapid stimulation of the MAPK activity, through both a PI3K- and MEK-dependent pathway. However, if the cells were previously treated for 5 min with 1 M okadaic acid, an effective inhibitor of MEK and MAPK through the inactivation of PP2A, a poor stimulation of MAPK was observed after vanadate treatment. Simultaneous addition of insulin and vanadate does not result in an additive effect, neither on MAPK nor in MEK, strongly suggesting that insulin and vanadate use the same signaling pathway from PI3K to MEK and MAPK [253, 254, 331]. There is only one study that reports possible inhibition of MAPK-specific tyrosine phosphatases by vanadium compounds. An oxovanadium glutamate complex, Na_2_[V(IV)O(Glu)_2_(CH_3_OH)]·H_2_O, showed potent inhibition against four human PTPs (PTP1B, TCPTP, HePTP, and SHP-1) with IC_50_ in the 0.21–0.37 μM ranges. However, only PTP1B presented a typical competitive inhibition. The vanadium inhibition mechanism for other phosphatases remains not clear [332]. It has been found that the proliferation of certain cells (chondrocytes VSa13 cells) is stimulated by vanadate through the activation of the MAPK pathway, even in the presence of wortmannin or PD98059 [333]. However, in fish preosteoblast cells, vanadate treatment did not stimulate cell proliferation through the MAPK pathway, but vanadate inhibited cell differentiation/ECM mineralization through the same mechanism that IGF-1 [333]. Decavanadate exhibited less efficiency than vanadate, but in longer treatments, similar effects were produced for both metavanadate and decavanadate solutions, stimulation of cell proliferation, and strong impairment (75%) of extracellular matrix (ECM) mineralization [334]. MAPK pathway in humans, animal models, or cells has been poorly studied; however, as it has been exposed, these pathways are strongly linked to insulin resistance, metabolic syndrome, cardiovascular diseases, and diabetes mellitus. Due to its effective activity as phosphatases inhibitor, it is possible that vanadate treatment stimulates both MEK and MAPK phosphorylation.

#### Vanadium: Inflammation and Redox Balance in Diabetes Mellitus

Vanadium compounds can interconvert into different species in living systems. This will occur primarily in the presence of reactive oxygen species and redox balance due to a Fenton mechanism [185]. Likewise, slight-generation of ROS is linked to the insulin signal transduction pathway [335]. Therefore, vanadium complexes could produce small amounts of ROS and enhance insulin signaling. The mimic insulin activity has been observed in some peroxovanadium complexes through ROS generation [14, 336–338]. However, if the ROS generation is too high, a decrease in insulin signaling might occur and its insulin mimic activity can be lost [336, 338, 339]. Vanadium itself may trigger oxidative stress at the cellular level, commonly by excessive administration of vanadium (higher than 5.0 mg/kg) [338–342]. Oxidovanadium (+ 4) and (+ 5) act as ROS generators such as peroxide, superoxide, hydroxyl radicals, and singlet oxygen [343]. Vanadate-dependent NADH oxidation associated with plasma membranes has been found to generate H_2_O_2_ [191]. Formation of H_2_O_2_ induced by vanadate has been shown to mediate apoptosis through the activation of p53 [344]. In p53-defective cells (tumor cells or non-tumor p53-knock out cells), vanadium compounds inhibit the cell cycle and induce apoptosis [345]. Activation of NF-κB by ROS generated by vanadium compounds enhances the apoptotic effect [346]. In contrast, in p53-functional cells, apoptosis is not shown [347].

Additionally, when cells or tissues are suffering from oxidative stress, MAPK can mediate the phosphorylation of nuclear factor erythroid 2 like 2 (Nrf2l2) and cause a disruption of Kelch-like ECH-associated protein 1 (Keap1) [348], which increases the expression of detoxifying enzymes, such as glutamate-cysteine ligase catalytic subunit (GCLC), heme oxygenase-1 (HO-1), and NAD(P)H quinine dehydrogenase 1 (NQO1) [349, 350], thus alleviating oxidative stress. Accumulating evidence indicate that vanadium compounds modulate the extent and duration of phosphorylation of some proteins, such as MEK-1, ERK 1/2, JNK, TNF-α, and NF-κB [251, 260, 351, 352], key effector proteins of the signaling pathways linked to the production of ROS and DNA damage. Activation of cell signaling pathways is mediated through regulating phosphorylation and dephosphorylation of proteins critical for signal transduction. Both, inactivation of phosphatases and activation of phosphokinases, lead to the generation of second messengers, the activation of downstream kinases. Inorganic salts of vanadium can activate phosphotyrosine phosphorylases of the ERK, c-Jun N-terminal kinase/stress-activated protein kinase (JNK/SARK), and p38, mainly by the oxidative stress increase, which is activated by a variety of stimuli and different cellular stresses such as insulin resistance, metabolic syndrome and diabetes mellitus [260, 351, 353, 354]. Both the ERKs and the JNK/SARK signaling pathways have also been implicated in NF-κB activation [355]. In type 1 diabetes mellitus NF-κB activity leads to β-cell dysfunction and death by apoptosis. Some studies have revealed that more than 66 genes are modified in the β-cell upon exposure to these cytokines. The activation of NF-κB can trigger pro- or anti-apoptotic cascades [356], but in β cells, the action is predominantly pro-apoptotic [357, 358]. NF-κB first became a chief suspect in the development of insulin resistance and type 2 diabetes after the milestone discovery that the anti-inflammatory agent, aspirin, inhibits NF-κB and prevents degradation of the NF-κB inhibitor, IκB [359, 360]. Although NF-κB is not directly involved, its participation is very important in metabolic disorders, because NF-κB is involved in increases in chronic liver inflammation, mimics high-fat diet or obesity-induced insulin resistance, and increases pro-inflammatory cytokines such as TNF-α, IL-1, and IL-6 levels, which are critical in the development of insulin resistance or survival cell signaling [361]. In this way, vanadium compounds such as bis(maltolato)-oxovanadium (IV) induce NF-κB nuclear translocation and apoptosis in B lymphocyte cell lineages but enhances the activation and survival of T cells [362]. Meanwhile, vanadate (V), vanadyl (VI), bis(maltolato)oxovanadium (IV), and bis(maltolato)dioxo-vanadium (V), all being promoters of MAPK and NF-κB, stimulated cell growth at low concentrations, but inhibited it at high concentrations, and induced distinct changes in cellular morphology, following overnight incubation. Bis(maltolato)dioxo-vanadium (V) is the least cytotoxic and the weakest inducer of morphological changes at low concentrations (10 μM), displaying a phosphorylation pattern similar to that of insulin [363]. Also, the bis-peroxovanadium (bpV), a potent PTPs inhibitor, activates NF-κB in human T lymphocytes without cell death [364]. Therefore, the results suggest that a balance between tyrosine kinases and tyrosine phosphatases establishes whether a cell will survive or undergo apoptosis. Moreover, the activation of cellular signaling pathways seems mainly to converge into NF-κB nuclear translocation and the transcription of either apoptotic (lethal) or anti-apoptotic genes. Researchers must provide substantial evidence for the chemical properties and biochemical effects of vanadium compounds in different cells or tissues in which vanadium has selective effects on metabolic control, survival, proliferation or apoptosis.

## Final Remarks

A large number of vanadium compounds have been synthesized and characterized as potential therapeutic agents for the treatment of diabetes mellitus, cancer, and diseases caused by parasites, viruses, and bacteria and are also proposed as anti-thrombotic, anti-hypertensive, anti-atherosclerotic, and spermicidal agents. In the present work, we focused on no transmissible chronic and degenerative diseases such as dysglycemia, dyslipidemia, insulin resistance, metabolic syndrome, and diabetes mellitus, because of its relevance worldwide. However, until now, no vanadium compound has proven to be efficacious for long-term use in humans, and only the bis(2-ethyl-3-hydroxy-4-pyronato)oxovanadium(IV) (BEOV) reached phase II in clinical trials. Consequently, the therapeutic dose of vanadium compounds is not well defined yet. The BEOV doses planned for a phase II clinical trial were 20 and 40 μg/day in patients with type 2 diabetes. Still, the minimal and maximal therapeutic dosage remain without being defined, which establishes a problem because of few works present an effective dose 50 (ED_50_), maximal dose, toxicological dose, and lethal dose which are key parameters to define the feasibility of a pro-drug could become a medicine. In animal models, the therapeutical dose in which vanadium acts as insulin-mimetic is high (0.5–1 mM). Importantly, the decavanadates show an important reduction in the level to a μM range. There are still many challenges in the use of vanadium compounds to treat diabetes. To date, not many investigations about action mechanisms, pharmacokinetics, pharmacodynamics, and posology have been generated. Likewise, the research model used in each study complicates the dosage, since each model possesses some features related to the metabolic diseases but not all of them. In this sense, the researchers must establish frameworks and restrictions if the model was induced by STZ, alloxan, high diets in carbohydrates or lipids, genetic modifications, or simply no diabetic animals. Some studies with vanadium compounds in cell cultures can help to elucidate with precision the potential sites of vanadium action. However, in vivo, because the microenvironment does not provide all metabolic pathways, toxicology, and detoxification between tissues, the effects observed are not necessarily the same. Additionally, the dosage used in cell cultures is not scalable because it would be in toxic ranges for its administration.

Another issue is that most of the time less than 5% of vanadium ingested orally is absorbed. In humans, it is estimated that only 0.13% to 0.75% of ingested vanadium is absorbed, while around 2.6% is absorbed in rats. Also, the effect of other dietary components, the form of vanadium in the stomach, and the speed at which it is transformed into V^4+^ probably affect the percentage of vanadium ingested/absorbed. In this sense, several works have demonstrated that vanadium compounds may result in other compounds or forms under different physiological conditions. Vanadium speciation is a matter of stability in biological media, due to synthetic chelators, biogenic ligands, or functional carriers. Thus, vanadium possesses a high ability to change oxidation states or ligands depending on the physiological environment (aqueous conditions, ligand concentration, pH, oxidative or reductive agents, and competition with other metals). However, most studies that approach the biological activity of vanadium compounds omit the possible speciation in the stomach, gut, and serum, as well as the concentration in blood of the original pro-drug. One way to solve this issue could be the use of nanomaterial-based platforms (nanomedicine) that could improve the dosage, selectivity, mechanism of action, posology, toxicological effect, etc., of vanadium-based therapeutics. Intracellular speciation is another critical point of the biological actions of vanadium. Vanadium speciation has been linked to redox balance and oxidative stress of cell and tissues. The redox properties of vanadium are determinant to its pharmacological effects because it can inhibit or stimulate proteins (mainly enzymes), traced back to what is termed “the vanadate–phosphate antagonism.” The enzymes most studied are phosphatases, such as PSPases and PTPases, but in metabolic diseases also affects glucose-6-phosphate dehydrogenase, nicotine adenine dinucleotide, adenosine diphosphate, nucleoside triphosphate diphosphohydrolases, phosphodiesterases, phosphoglucomutases, and ATPases. PTP1B has been deeply studied since this enzyme is directly involved in the insulin signaling and vanadium compounds are competitive inhibitors, which makes it a potential therapeutical target. Furthermore, as was exposed, insulin has multiple biological actions and different convergence pathways with other hormones, biogenic peptides, neurotransmitters, cytokines, and interleukins. Therefore, to study the vanadium activity on PTPB1 is just a “reductionist” way since there are multiples options for the inhibition or activation of vanadium on kinases and phosphatases that take part in the metabolic and signaling cascades routes.

Although there are skeptical views on the role of vanadium compounds in the treatment of diabetes, mainly due to long-term toxicity [35], it is, without doubt, a fascinating field of research.

Finally, the investigations into vanadium chemistry (after almost 200 years of its discovery) are not completely understood. From the biological perspective, we know that vanadium compounds have a great potential in the treatment of many types of diseases. However, we must first understand in detail the mechanisms of transport, therapeutical targets, pharmacokinetics, and pharmacodynamics, for the better and more efficient design of the vanadium-based drugs.

## Abbreviations

ACC: Acetyl CoA carboxylase
Akt/PKB: Protein kinase B
ATSDR: Agency for toxic substances and disease registry
AS160: Akt substrate of 160 kDa
BAD: Bcl-2-associated death promoter
Bax: Bcl-2-like protein 4
Bcl-xL: B cell lymphoma-extra large
BEOV: Bis(ethylmaltolato)oxovanadium(IV)
BMOV: Bis(maltolato)oxovanadium (IV)
ChREBP: Carbohydrate response element-binding protein
CREB: cAMP response element-binding
Cyc D1: Cyclin D1
DAG: Diacylglycerol
DMT1: Divalent metal transporter-1
ELOVL6: Elongation of long-chain fatty acids family member 6
ERK: Extracellular signal-regulated kinases
F2,6P: Fructose-2,6-bisphosphate
F6P: Fructose-6-phosphate
FAS: Fatty acid synthase
FFA: Free fatty acid
FOXO: Forkhead box O
G6P: Glucose-6-phosphate
GLUT2: Glucose transporter type 2
GLUT4: Glucose transporter type 4
GRB2: Growth factor receptor-bound protein 2
GS: Glycogen synthase
GSK3β: Glycogen synthase kinase 3β
IgG: Immunoglobulin G
IRS1: Insulin receptor substrate 1
JNK: c-Jun N-terminal kinases
LPA: Lysophosphatidic acid
LXRα: Liver X receptor α
LOAEL: Lowest-observed-adverse-effect level
MAPK: Mitogen-activated protein kinase
MBS: Multimetal binding site
MCT1: Monocarboxylate transporter
MEK: Mitogen-activated protein kinase kinase
MRL: Minimal risk level
mTORC1: Mammalian target of rapamycin complex 1
mTORC2: Mammalian target of rapamycin complex 2
NBS: N-terminal site
NOAEL: No-observed-adverse-effect level
OCT: Organic anion transporter
p38: p38 mitogen-activated protein kinases
PA: Phosphatidic acid
PDK1: Phosphoinositide-dependent kinase 1
PFK2: Fosfofructoquinasa-2
PHLPP: Pleckstrin homology domain leucine-rich repeat protein phosphatase
PI3K: Phosphoinositide 3-kinase
PIP2: Phosphatidylinositol (4,5)-bisphosphate
PIP3: Phosphatidylinositol (3,4,5)-trisphosphate
PKCε: Protein kinase C epsilon isoform.
PKCθ: Protein kinase C theta isoform
Pklr: Pyruvate kinase isozymes R/L
PTEN: Phosphatase and tensin homolog deleted on chromosome 10
PTPases: Tyrosine-phosphate proteins
PTP1B: Protein tyrosine phosphatases 1B
PSPases: Serine-threonine phosphate proteins
Raf: Rapidly accelerated fibrosarcoma
Ras: Transforming protein p21
Rheb: Ras homolog enriched in brain
S6K: Ribosomal protein S6 kinase
SCD1: Stearoyl-CoA desaturase 1
SDT: Saturation transfer difference
SHC: Src homology 2 domain-containing
SOS: Son of sevenless homolog 1
STEAP: STEAP metalloreductase
STZ: Streptozotocin
SREBP1c: Sterol regulatory element-binding protein 1c
TSC1–TSC2: Hamartin–tuberin complex
VBS1: High-affinity vanadium binding site
VBS2: Low-affinity vanadium binding site
VLDL: Very low-density lipoprotein
